# The Trophoblast Compartment Helps Maintain Embryonic Pluripotency and Delays Differentiation towards Cardiomyocytes

**DOI:** 10.3390/ijms241512423

**Published:** 2023-08-04

**Authors:** Xiang Zhao, Bethany N. Radford, Mark Ungrin, Wendy Dean, Myriam Hemberger

**Affiliations:** 1 Department of Cell Biology and Anatomy, Cumming School of Medicine, University of Calgary, 3330 Hospital Drive NW, Calgary, AB T2N 4N1, Canada; zhaox@ucalgary.ca; 2 Alberta Children’s Hospital Research Institute, University of Calgary, Calgary, AB T2N 4N1, Canada; bethany.radford@ucalgary.ca (B.N.R.); mdungrin@ucalgary.ca (M.U.); 3 Department of Biochemistry and Molecular Biology, Cumming School of Medicine, University of Calgary, 3330 Hospital Drive NW, Calgary, AB T2N 4N1, Canada; 4 Faculty of Veterinary Medicine, University of Calgary, 3330 Hospital Drive NW, Calgary, AB T2N 4N1, Canada

**Keywords:** embryonic stem cells, trophoblast stem cells, cardiomyocyte differentiation, embryoid bodies, mesoderm differentiation

## Abstract

Normal developmental progression relies on close interactions between the embryonic and extraembryonic lineages in the pre- and peri-gastrulation stage conceptus. For example, mouse epiblast-derived FGF and NODAL signals are required to maintain a stem-like state in trophoblast cells of the extraembryonic ectoderm, while visceral endoderm signals are pivotal to pattern the anterior region of the epiblast. These developmental stages also coincide with the specification of the first heart precursors. Here, we established a robust differentiation protocol of mouse embryonic stem cells (ESCs) into cardiomyocyte-containing embryoid bodies that we used to test the impact of trophoblast on this key developmental process. Using trophoblast stem cells (TSCs) to produce trophoblast-conditioned medium (TCM), we show that TCM profoundly slows down the cardiomyocyte differentiation dynamics and specifically delays the emergence of cardiac mesoderm progenitors. TCM also strongly promotes the retention of pluripotency transcription factors, thereby sustaining the stem cell state of ESCs. By applying TCM from various mutant TSCs, we further show that those mutations that cause a trophoblast-mediated effect on early heart development in vivo alter the normal cardiomyocyte differentiation trajectory. Our approaches provide a meaningful deconstruction of the intricate crosstalk between the embryonic and the extraembryonic compartments. They demonstrate that trophoblast helps prolong a pluripotent state in embryonic cells and delays early differentiative processes, likely through production of leukemia inhibitory factor (LIF). These data expand our knowledge of the multifaceted signaling interactions among distinct compartments of the early conceptus that ensure normal embryogenesis, insights that will be of significance for the field of synthetic embryo research.

## 1. Introduction

Early development in the mouse is characterized by the separation of the embryonic and trophoblast lineages that are set aside in the first cell fate decision event after fertilization. This differentiation event results in the emergence of the outer trophectoderm (TE) and the inner cell mass (ICM) of the blastocyst stage embryo. These cell populations go on to form the extraembryonic ectoderm (ExE) and epiblast (EPI) compartments, respectively, of the egg cylinder stage embryo after implantation. Cells of the primitive endoderm (PE) lineage form the visceral endoderm (VE) that overlies the EPI and is also predominantly extraembryonic in fate [1]. Despite the clear cell fate separation and lineage commitment of these cell populations, the EPI, ExE and VE compartments remain interconnected, firstly at their adjoining interfaces where cells are in direct contact with each other, and secondly through connection via the proamniotic canal that spans the EPI and ExE compartments. Hence, crosstalk between these cell populations remains possible and is, indeed, vital for the normal progression of development.

The interdependence and interconnectivity among these early cell lineages have been demonstrated on multiple levels. Perhaps the best-documented example is that of the anterior visceral endoderm (AVE) that signals to the underlying EPI to establish distinct gene expression domains instructing the patterning of the anterior pole of the embryo [2]. Similarly, bone morphogenetic protein 4 (BMP4) is a key ExE-produced growth factor that acts on the posterior pole of the embryo where it sets up a unique niche within the emerging allantoic bud required for the formation of primordial germ cells [3]. Another example is that of NODAL, a TGFb superfamily member involved in anterior–posterior and left–right patterning of the embryo. Although produced in a pro-form by the embryo itself, the enzymes necessary to generate the active form of NODAL are expressed by the ExE, which thereby contributes to the establishment of growth factor gradients necessary for regional specification [4,5].

These examples highlight the pivotal role of extraembryonic tissues in embryo patterning and axis determination. Conversely, EPI-produced factors, notably fibroblast growth factor 4 (FGF4), are critical to maintain the proliferative and self-renewal capacity of the ExE’s trophoblast. In fact, the reliance of ExE proliferation and expansion on FGF signals was realized through a series of mouse mutants targeting the FGF-mitogen activated protein kinase (MAPK) pathway [6]. These data ultimately guided strategies into the derivation of trophoblast stem cells (TSCs) that can be maintained in a self-renewing state in the presence of exogenous FGF [7].

It is in this environment of reciprocal tissue interactions when the precursors of the first heart anlagen are specified. The future heart develops from cardiogenic mesodermal progenitors positioned just posterior to the head fold. At embryonic day (E)6.5, these progenitors ingress through the primitive streak and migrate away from it to form the heart fields [8]. Later additional contributions to the heart derive from the proepicardium and cardiac neural crest cells. Yet, it is the cardiogenic mesoderm and cardiac crescent formation that takes place in the peri-gastrulation embryo where close proximity to extraembryonic tissues of the VE and ExE allows for potential induction signals to occur. It stands to reason that such early interactions may explain why several gene mutations that cause mid-gestational lethality, such as those for *Pparg*, *Mapk14* (*p38a*), *Mapk1* (*Erk2*) or *Senp2*, display cardiac defects that can be rescued solely by providing the embryo with a functional trophoblast compartment [9,10,11,12].

Here, we tested the hypothesis that trophoblast cells equivalent to those found in the ExE affect early cardiogenesis. This goal was accomplished by deconstructing the lineage components of the post-implantation conceptus in an in vitro system. Using mouse embryonic stem cells (ESCs) carrying an alpha-cardiac myosin heavy chain reporter transgene (*Myh6*-GFP) for optimized embryoid body formation protocols reporting the differentiation into beating cardiomyocytes, we show that trophoblast-conditioned medium normally impedes the cardiomyocyte differentiation process at the stage of cardiac mesoderm formation. We also demonstrate that conditioned medium from TSCs mutant for genes that affect the trophoblast–heart axis differentially interferes with this normal trajectory. Overall, our data indicate that trophoblast secretions prolong the pluripotent state of ESCs and slow down the epithelial–mesenchymal transition (EMT) that is requisite for mesodermal differentiation. This effect is likely mediated through the potent production of leukemia inhibitory factor (LIF) by TSCs. These findings extend our knowledge of the various crucial interactions between the distinct cell lineages of the early conceptus, and highlight that the crosstalk between EPI and ExE is indeed reciprocal for the maintenance of cellular plasticity in both compartments.

## 2. Results

### 2.1. Establishment of a Robust Differentiation Protocol of Murine ESCs into Cardiomyocytes

Suspension culture of mouse ESCs in non-adherent cell culture dishes is a well-established method to promote formation of embryoid bodies (EBs) that recapitulate developmental processes reminiscent of early embryonic development, including the differentiation into derivatives of the three germ layers, endo-, meso- and ectoderm [13]. One of the visually most intriguing outcomes is the differentiation of cardiomyocytes over an 8-day time course, culminating in beating EBs [14,15]. Despite the long-standing use of EBs in in vitro differentiation protocols, this method is prone to notorious variability in each experimental set-up caused by inconsistent initial cell plating numbers [16] that drive differences in the size and shape of forming EBs, and by uncontrolled coalescence among individual EBs into larger aggregates.

Here, we optimized a culture protocol using size-controlled EBs formed in microwells, with a controlled initial cell seeding density of 600 cells per microwell [17]. The aggregation of ESCs in the microwell was achieved by gentle centrifugation [18]. After 2 days (D2) of culture, the cell aggregates were transferred into a suspension culture in bacteriological Petri dishes and maintained on a rotary orbital shaker at 25 rpm and a tilt angle of 4.5 degree. On D4, EBs were either left in suspension culture for observation, or for molecular analyses plated back on gelatin-coated tissue culture dishes at a defined EB density (15 EBs/24-well) to promote attachment and cardiomyocyte differentiation (Supplementary Materials Figure S1A). This method achieved extremely uniform EB sizes within each successive experimental set-up and among different rounds of experiments (Supplementary Materials Figure S1B,C) and resulted in highly reproducible differentiation rates and marker gene activation dynamics.

### 2.2. Generation of Trophoblast-Conditioned Medium (TCM)

**Figure 1:**
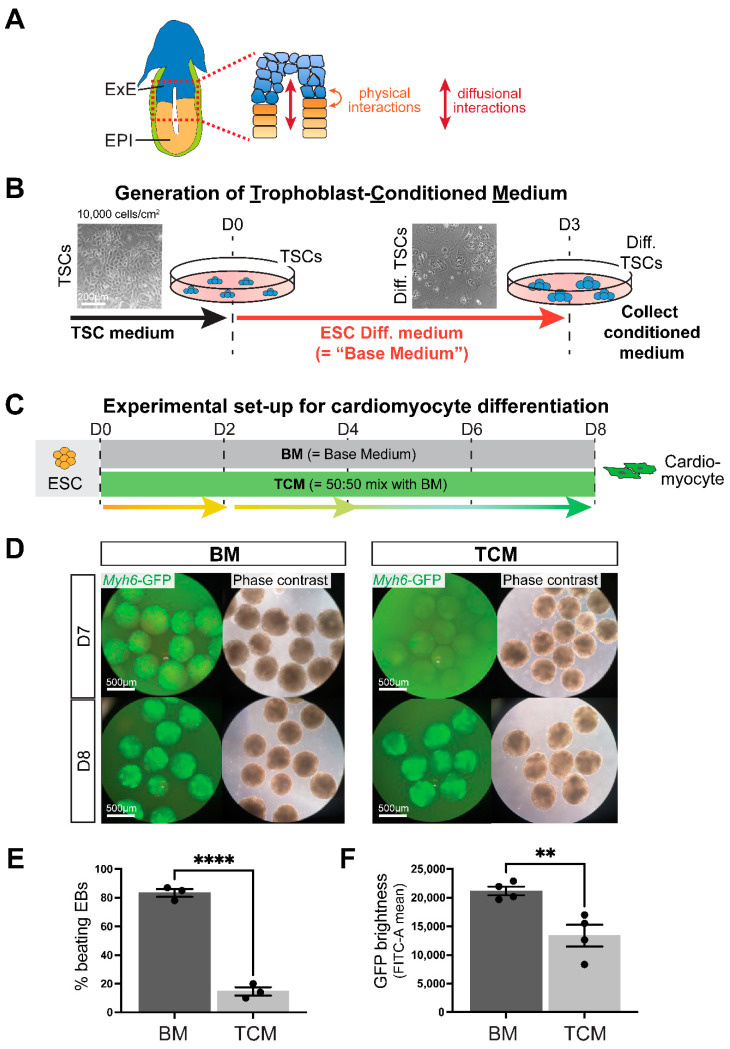
Trophoblast-conditioned medium affects cardiomyocyte differentiation from ESCs. (**A**) Diagram of an E6.5 mouse conceptus with a magnified view of the embryonic-trophoblast interface enabling possibilities for crosstalk. EPI = epiblast, ExE = extra-embryonic ectoderm. (**B**) Schematic depiction of the generation of trophoblast-conditioned medium (TCM). Trophoblast stem cells (TSCs) were exposed to ESC cell differentiation medium, also referred to as “base medium” (BM) that does not contain any growth factor supplements, for three days. Because of the absence of FGF, TSCs start to differentiate during this time. (**C**) Experimental set-up for cardiomyocyte differentiation time course from embryonic stem cells (ESCs) in either base medium (BM) or media consisting of 50% TCM: 50% BM, for brevity referred to as “TCM” in the subsequent figures. (**D**) Cardiomyocyte differentiation in embryoid bodies visualized by a cardiac-specific α-myosin heavy chain (MHC, *Myh6*) promoter-driven enhanced green fluorescent protein (GFP) construct shows the appearance of GFP-positivity at day 7 (D7) in BM but only at D8 in TCM. For visualization purposes, EBs were cultured in suspension culture from D2 onward. Data are representative of 12 independent biological replicates. (**E**) Percentage of beating embryoid bodies (EBs) at D8 of differentiation in EBs grown in suspension culture from D2 onward in BM or TCM. **** *p* < 0.0001, two-tailed Student’s *t*-test. (**F**) GFP intensity measured by flow cytometry as FITC-A mean in EBs at D10 of differentiation in BM or TCM. Data are displayed as the mean ± SEM. Statistical analysis was conducted by unpaired two-tailed Student’s *t*-test. ** *p* < 0.01.

In the early post-implantation conceptus, only very few cells of the ExE cells are in direct contact with the EPI (Figure 1A). Therefore, we reasoned that any putative interaction between both compartments is likely mediated through the production and secretion of growth factors or growth factor modulators that can have paracrine action. To test this hypothesis, we cultured mouse TSCs in standard ESC differentiation medium (15% serum in DMEM, see Materials and Methods, termed “base medium” (BM)) for three days without media change (Figure 1B). Since BM lacks FGF4, an essential growth factor for TSC maintenance, the TSCs will start to differentiate over this 3-day time period. Therefore, the medium is first exposed to trophoblast stem cells followed by early differentiated trophoblast cells, representative of the cell population present in the ExE. We collected the resulting trophoblast-conditioned medium (TCM) after three days and used it as a 50:50 mix with BM in all downstream experiments (Figure 1B).

### 2.3. TCM Slows down Cardiomyocyte Differentiation Dynamics

Following the EB differentiation protocol established above, we then carried out ESC differentiation experiments towards cardiomyocytes either in regular BM or in 50% TCM (Figure 1C). We verified that the TCM media was not pH-changed or otherwise depleted for supplements, an aspect further ascertained by the 50:50 media mixing procedure. Early cell aggregates at D3 were of the same size in BM and TCM, but over subsequent days TCM-exposed EBs expanded more slowly and did not form a fluid-filled cavity as quickly as in BM, resulting in smaller EBs at D4 and D5. By D6, TCM-grown EBs started to catch up with BM controls, and were of the same size by D7 and D8 (Supplementary Materials Figure S2A). 

To monitor the dynamics of cardiomyocyte differentiation in live culture we used ESCs carrying an alpha-cardiac myosin heavy chain reporter transgene (*Myh6*-GFP) that results in GFP expression in maturing cardiomyocytes. In standard BM differentiation conditions, GFP-positive cells were routinely first detected by D7, and their numbers and GFP intensity increased steadily over subsequent days of differentiation. In stark contrast, in the presence of 50% TCM, no or only very few GFP-positive cells were observed on D7 (Figure 1D, Supplementary Materials Figure S2B). By D8, the TCM-exposed EBs had started to express the *Myh6*-GFP transgene, but the number of beating EBs was still much reduced compared to cells grown in BM (Figure 1E, Supplementary Materials Videos S1 and S2). Even though the cardiomyocyte differentiation trajectory seemed to catch up at later stages of the differentiation time course based on expression of the mature cardiomyocyte markers cardiac troponin T2 (*Tnnt2*) and *Myh6* (Supplementary Materials Figure S2C), the GFP intensity of TCM-grown EBs and the frequency of beating EBs remained reduced even on D9-10 (Figure 1F, Supplementary Materials Figure S2D). 

Overall, these results demonstrated that trophoblast cells produce compounds that serve to slow down cardiomyocyte differentiation from pluripotent precursors.

### 2.4. TCM Interferes Specifically with the Early Phases of Mesoderm and Cardiac Mesoderm Formation

**Figure 2:**
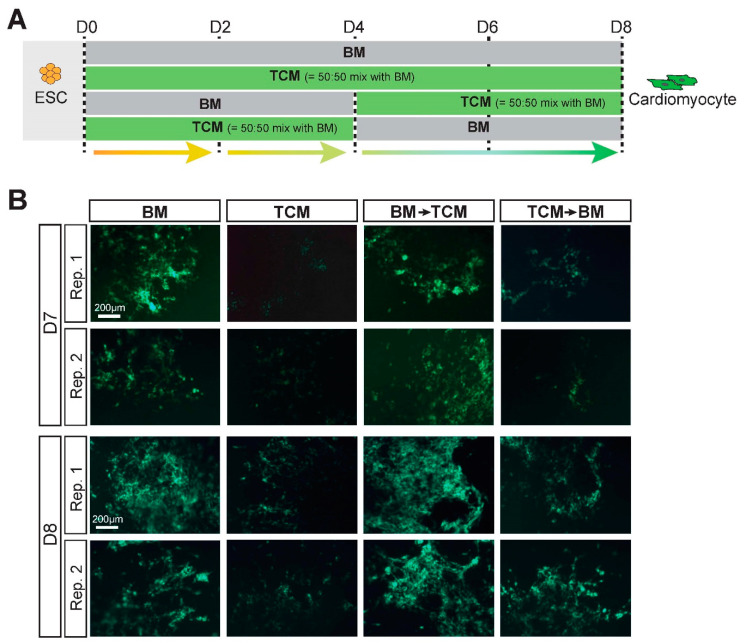
TCM impacts the first phase of ESC differentiation towards cardiomyocytes. (**A**) Experimental layout of the media-switch experiment between BM and TCM at D4. (**B**) Appearance of *Myh6*-reporter driven GFP-positive cells at D7 and D8 of the differentiation time course in EBs grown in adhesion culture from D4 onward. EBs exposed only for the first 4 days to TCM (“TCM -> BM”) exhibit almost the same delay in cardiomyocyte differentiation as those cultured in TCM for the entire duration. Data are representative of 3 independent biological replicates.

**Figure 3:**
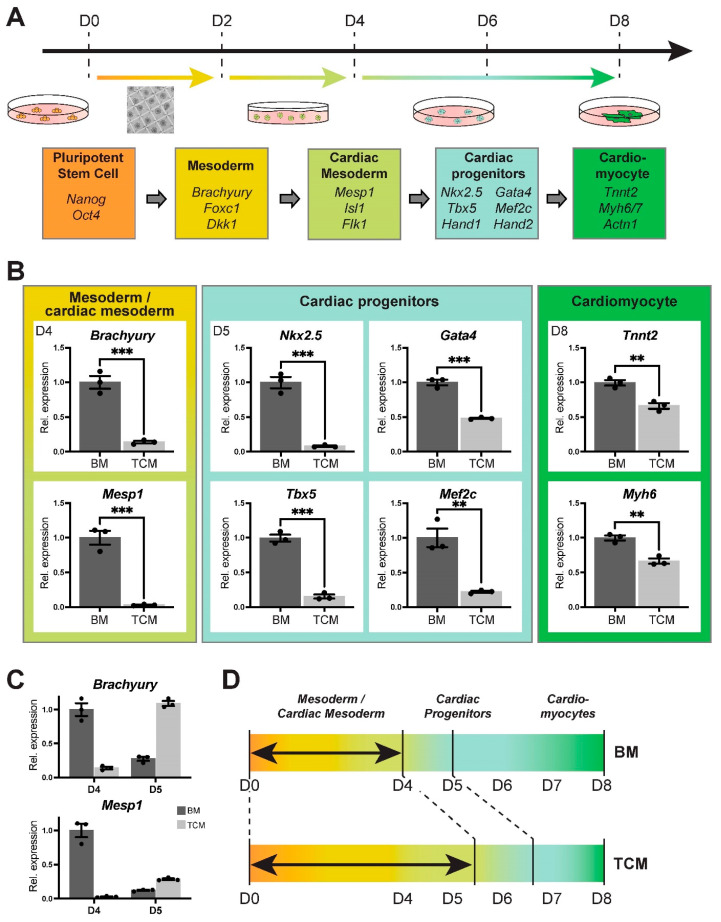
TCM delays mesoderm and specifically cardiac mesoderm differentiation. (**A**) Schematic depiction of the progression of differentiation from ESCs to cardiomyocytes including representative marker genes. (**B**) RT-qPCR analysis of differentiation stage-specific marker genes representative at various days of differentiation, as indicated. The most affected processes are the specification of cardiac mesoderm (*Mesp1*) at D4 and of cardiac progenitors (*Nkx2.5*, *Tbx5*) at D5. Data of three independent biological replicates are normalized to BM conditions and are displayed as mean ± SEM. Statistical analysis was by unpaired two-tailed Student’s *t*-test. ** *p* < 0.01, *** *p* < 0.001. (**C**) Progression of mesoderm (*Brachyury*) and cardiac mesoderm (*Mesp1*) specification at D4 and D5 of differentiation in EBs from the same starting samples. In BM-grown EBs, *Brachyury* peaks at D4 and has declined in expression levels at D5. In TCM, *Brachyury* reaches peak expression only at D5. *Mesp1* activation is even more delayed. (**D**) Diagrammatic depiction of the time frame most impacted by TCM.

Our next experimental approaches were aimed at determining the precise steps in the ESC-to-cardiomyocyte differentiation process that are impacted by TCM. To narrow down the possibilities, we performed a media swap experiment exposing the EBs to TCM either only for the first four days (D0–D4), or only for the latter four days (D4–D8), of the differentiation protocol (Figure 2A). These data revealed that the greatest impact of TCM was during the first four days, delaying the onset of *Myh6*-driven GFP reporter fluorescence and the frequency of beating EBs as profoundly as when the EBs were grown in TCM for the entire duration (Figure 2B, Supplementary Materials Figure S2D). We also performed gene expression analysis for differentiation stage-specific marker genes on EBs cultured in BM and TCM, and found that the strongest effect of TCM was on the emergence of mesoderm and specifically cardiac mesoderm progenitors (Figure 3A,B, Supplementary Materials Figure S3). Thus, we observed a severe impact on the induction of mesoderm marker *Brachuyry* and cardiac mesoderm marker *Mesp1* at D4 and *Flk1* at D5, and consequently on cardiac progenitor markers such as *Nkx2.5*, *Tbx5*, *Gata4* and *Mef2c* at D5 (Figure 3B, Supplementary Materials Figure S3B). While mature cardiomyocyte markers at D8 were still not as highly expressed in TCM-exposed EBs compared to EBs grown in BM, the discrepancy in expression was less marked than for cardiac progenitor markers at earlier stages (Figure 3B), and levelled out with controls by D10 (Supplementary Materials Figure S2C). Moreover, the expression of cardiac progenitor markers was similar in BM and TCM conditions at D8, implying that TCM-exposed EBs were able to catch up in their cardiomyocyte differentiation trajectory during the later stages of the differentiation time course (Supplementary Materials Figure S3C). To examine the transition through the apparent bottleneck stages around D4-D5 in more detail, we compared *Brachuyry* and *Mesp1* expression in EBs from the same starting pools. In BM-grown EBs, both genes peaked at D4 and declined at D5; by contrast, TCM-grown EBs exhibited peak expression of *Brachyury* only at D5 and *Mesp1* induction followed from D5 onwards (Figure 3C, Supplementary Materials Figure S3B,D). These data implied that TCM causes a differentiation delay specifically during the early stages of mesoderm and cardiac mesoderm formation (Figure 3D).

### 2.5. Expression Profiling Underscores the Impact of TCM on Mesoderm Differentiation

**Figure 4:**
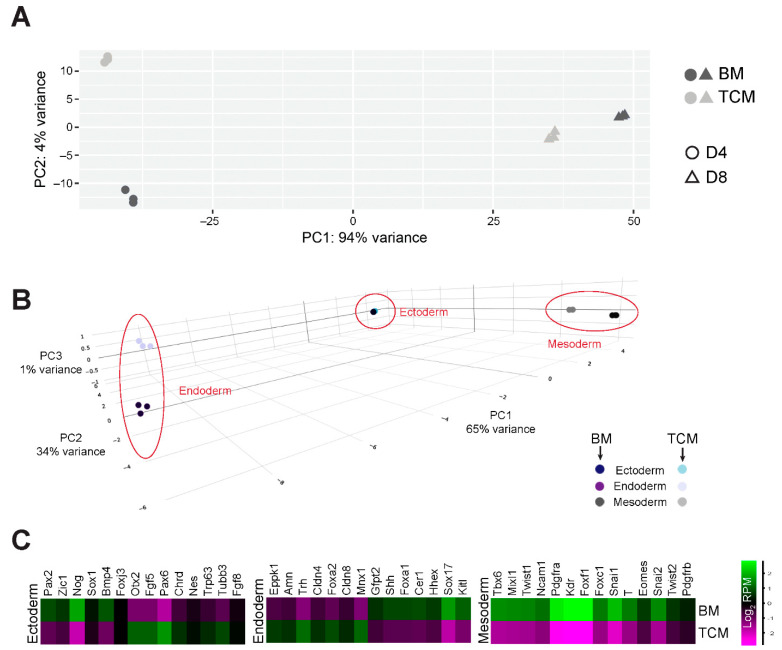
RNA expression profiling of BM- and TCM-exposed EBs. (**A**) Principal component analysis (PCA) of top 500 most highly expressed genes of D4 and D8 EBs grown in BM and TCM. The greatest variance is introduced by day of differentiation, as expected. However, principal component 2 (PC2) distinguishes BM from TCM effects and shows a greater divergence of samples at D4 compared to D8. (**B**) 3-dimensional PCA plot of D4 EBs based on germ layer lineage marker expression. Mesoderm markers separate TCM- from BM-grown EBs along PC2 which accounts for 34% of variance, whereas endoderm markers separate these samples only along PC3 with 1% variance. Ectoderm markers do not separate the samples. (**C**) Heatmaps of gene expression levels of selected germ layer lineage markers in D4 EBs cultured in TCM vs. BM. The greatest divergence is seen for mesoderm markers that are collectively and uniformly down-regulated in TCM conditions, as indicated by the brightest colour intensity differences compared to markers of the endoderm and ectoderm lineages.

To assess the impact of TCM on ESC-to-cardiomyocyte differentiation on the global level, we performed RNA-seq on BM-control and TCM-exposed EBs at D4 and D8. The overall sample similarity between independent replicate experiments was extremely tight, confirming the robustness and reproducibility of our EB differentiation protocol (Supplementary Materials Figure S4A). Principal component analysis (PCA) revealed that the greatest difference between these samples was determined by differentiation stage (PC1), as expected. However, PC2 separated the samples by culture media, and demonstrated a higher degree of divergence between BM- versus TCM-grown EBs at D4 compared to D8 (Figure 4A). These data confirmed that the greatest impact of TCM was on early stages of differentiation.

Differential gene expression analysis at D4 identified 2815 up- and 1112 down-regulated genes in TCM vs. BM samples (Supplementary Materials Figure S4B). Interestingly, gene set enrichment analysis revealed terms specifically related to coronary and heart morphogenesis as depleted in the TCM-grown EBs (Supplementary Materials Figure S4C). The transcriptome-wide readout of the RNA-seq data also resolved the question whether the TCM-mediated differentiation inhibition was limited to mesoderm or whether it also affected the other germ layer lineages, i.e., endo- and ectoderm. Overall, our culture conditions were compatible with differentiation into all three lineages, as expected (Supplementary Materials Figure S4D) [19]. Comparing the progress of differentiation at D4 between BM and TCM, ectoderm differentiation was not or only minimally affected by TCM (Figure 4B, Supplementary Materials Figure S4D). Endoderm markers also showed a much more nuanced and inconsistent behaviour with some up- and some down-regulated genes (Figure 4B,C, Supplementary Materials Figure S4D). Amongst these, however, we noted that *Hhex* and *Sox17* were significantly down-regulated (Supplementary Materials Figure S3E), i.e., genes that are critical for cardiac mesoderm formation from ESCs [20]. Yet the by far most consistent effect overall was on mesoderm markers that were globally not induced to the same level in D4 TCM- compared to BM-cultured EBs (Figure 4B,C, Supplementary Materials Figure S4D).

Collectively, these data showed that the TCM-induced slowdown in ESC differentiation towards cardiomyocytes specifically occurs during early stages and impedes the formation of mesoderm, cardiac mesoderm and cardiac progenitors.

### 2.6. TCM from Mutant Trophoblast Stem Cells Differentially Affects Cardiac Differentiation Dynamics 

We then asked whether the trophoblast-mediated effect was responsive to ablation of certain genes in the TSCs used for TCM generation. To test this possibility, we chose 4 different mutant TSC lines ablated for *Ssr2*, *Atp11a*, *Smg9* and *Pparg*. These gene knockouts (KOs) were chosen for the following reasons: All of them affect placenta and heart development in mouse mutants, however, the cardiac abnormalities in the *Ssr2* mutants are independent of placental defects, whereas those in *Atp11a* mutants are entirely due to placental defects, manifesting after mid-gestation between E12.5–E14.5 [21]. *Smg9* mutants exhibit shared contributions from the embryonic and trophoblast compartment to the etiology of heart defects [21]. Cardiac development in *Smg9*-null embryos is already severely affected by E10.5 with profound outflow tract and ventricle formation defects (Supplementary Materials Figure S5A). Finally, the *Pparg* mutation is the paradigm example that evoked the concept of the placenta–heart axis; mutants exhibit severe heart abnormalities that, like in *Atp11a* mutants, are secondary to placental defects but manifest earlier with embryonic lethality occurring around E10-10.5 [9]. Thus, KO TSCs for these genes provided a comprehensive spectrum for modelling trophoblast-induced heart defects in vitro. 

**Figure 5:**
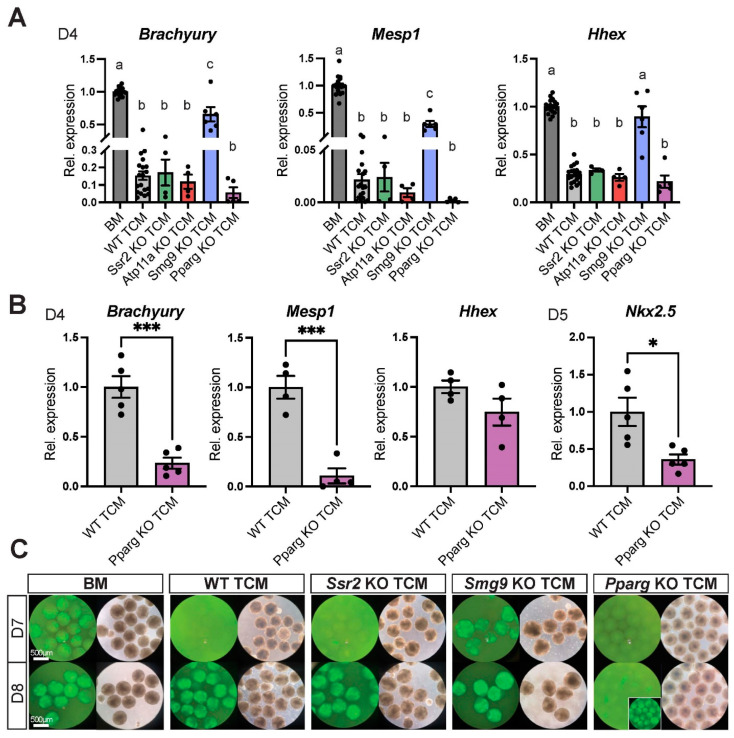
TCM from TSCs mutant for placenta–heart axis genes affects the dynamics of EB differentiation. (**A**) RT-qPCR analysis of EBs at D4 grown in BM or TCM from wild-type (WT) or various mutant TSCs. Gene mutations that have an early impact on cardiogenesis in vivo (*Smg9*, *Pparg*) alter EB differentiation rates compared to WT TCM. By contrast, conditioned medium from TSCs mutant for genes that either do not have a trophoblast effect on heart development in vivo (*Ssr2*) or that exhibit a later gestational impact of the placenta on heart formation (*Atp11a*) have no divergent effect from WT TCM. *Smg9* knockout (KO) TCM releases the differentiation delay, whereas *Pparg* KO TCM seemingly exacerbates it. Data are representative of four biological replicates per KO TCM, each performed with their internal BM and WT TCM controls. Values are normalized to BM and are displayed as mean ± SEM. Statistical analysis was conducted using one-way ANOVA with Tukey’s multiple comparisons test. The letters above bars denote the outcome of statistical comparisons, with statistically significant differences (*p* < 0.05) indicated by discrepant letters, whereas identical letters indicate *p* > 0.05. (**B**) Comparative analysis of the effects of *Pparg* KO TCM vs. WT TCM on EB differentiation dynamics assessed by RT-qPCR for cardiac-informative marker genes at D4 and D5, as indicated. *Pparg* KO TCM further exacerbates the differentiation-inhibiting effect seen with WT TCM. Data are of 4–5 independent biological replicates normalized to WT TCM conditions and are displayed as mean ± SEM. Statistical analysis was performed using unpaired two-tailed Student’s *t*-test. * *p* < 0.05 and *** *p* < 0.001. (**C**) *Myh6*-GFP reporter gene activation as a readout of cardiomyocyte differentiation in non-adherent EBs at D7 and D8 of differentiation. For visualization purposes, EBs were cultured in suspension culture from D2 onward. *Smg9* KO TCM accelerates differentiation with EBs becoming GFP-positive already at D7, similar to what is seen with BM. *Pparg* KO TCM further delays the onset of GFP fluorescence beyond what is seen with WT TCM or with *Ssr2* and *Atp11a* KO TCM. The inset shows EBs at D9 when they have turned green.

We generated TCMs from each of these KO TSC lines using multiple independent KO clones, as well as from their corresponding wild-type (WT) counterpart TSC clones. When applying these KO TCMs (again, mixed 50:50 with BM) to the ESC-to-cardiomyocyte differentiation protocol, we found that the differentiation delaying capacity of *Ssr2* KO TCM and *Atp11a* KO TCM was indistinguishable from that of WT TCM control, with strong effects on mesoderm (*Brachyury*), mes-endoderm (*Hhex*) and in particular on cardiac mesoderm (*Mesp1*) differentiation (Figure 5A, Supplementary Materials Figure S5B) [22]. By contrast, *Smg9* KO TCM had significantly lost this inhibitory capacity, with endoderm differentiation occurring normally and mesoderm/cardiac mesoderm differentiation much less affected, almost resembling the dynamics observed in BM. This observation also served to rule out that the observed TCM effects were somehow related to media depletion, a point further offset by the 50:50 mixture of TCM with BM. 

Conversely, *Pparg* KO TCM appeared to have an even more pronounced inhibitory effect than WT TCM (Figure 5A). To assess this latter point in more detail, we used WT TCM and *Pparg* KO TCM in side-by-side experiments, and indeed observed an even more exacerbated inhibition of differentiation into mesoderm (*Brachyury*), cardiac mesoderm (*Mesp1*) and cardiac progenitors (*Nkx2.5*) with *Pparg* KO TCM (Figure 5B, Supplementary Materials Figure S5C). These altered differentiation dynamics were consistently detected also with the *Myh6*-GFP reporter at D7 and D8 in EBs kept in suspension culture. At D7, EBs grown in *Smg9* KO TCM already appeared bright green similar to EBs cultured in BM, whereas WT TCM-grown EBs did not (Figure 5C). *Pparg* KO TCM-exposed EBs were still not green at D8 unlike those in all other conditions, and only eventually became GFP-positive as indicator of mature cardiomyocyte differentiation at D9 (Figure 5C, inset). 

These data showed that the cardiomyocyte differentiation system is sensitive to the genetic make-up of the TSCs from which the TCM is derived. More importantly even, the cardiomyocyte differentiation dynamics are altered specifically in response to gene mutations that have an early trophoblast-induced effect on cardiogenesis in vivo.

### 2.7. TCM Promotes ESC Pluripotency

**Figure 6:**
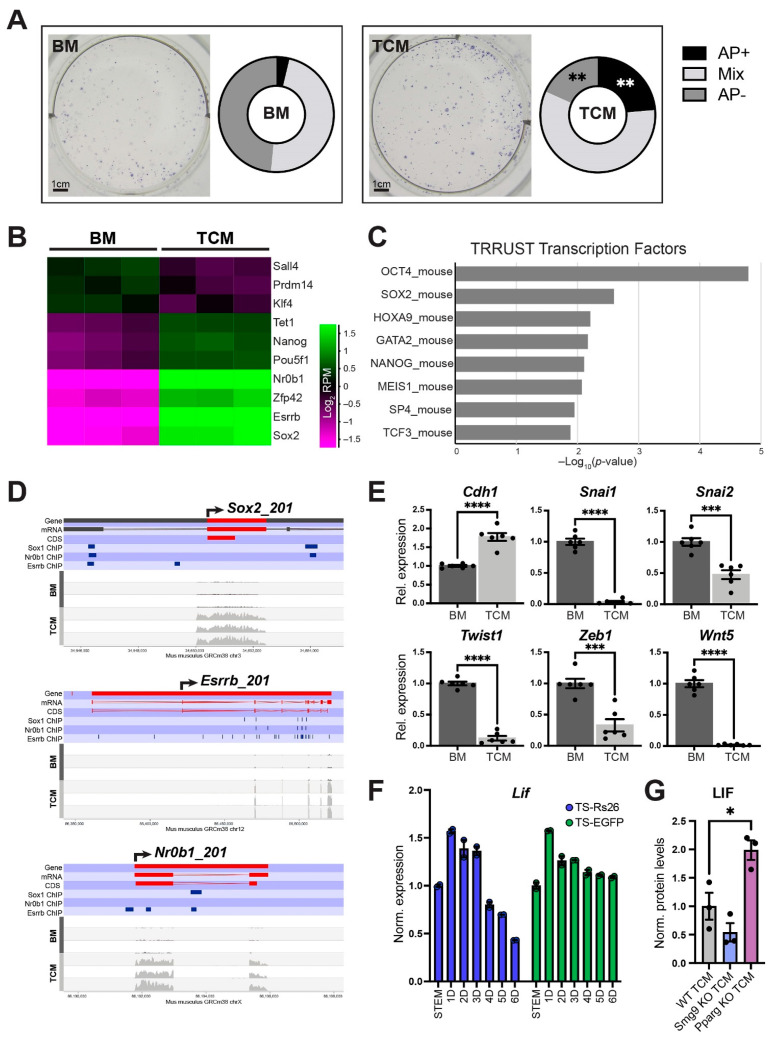
TCM prolongs the expression of pluripotency features in ESCs and EBs. (**A**) Alkaline phosphatase (AP) staining as pluripotency marker in ESCs grown for 5 days in BM or TCM. The number of AP-positive colonies is significantly higher in TCM conditions. Data are from three independent biological replicates. Statistical analysis was by multiple unpaired Student’s *t*-tests with Holm-Šídák multiple comparisons test. ** *p* < 0.01. (**B**) Heatmap of normalized Log_2_-transformed RNA-seq read counts of 10 selected, key pluripotency genes that are differentially expressed between D4 EBs grown in BM vs. TCM. The majority of these genes retains higher expression levels in TCM conditions, with the most pronounced differences (>8-fold) observed for *Sox2*, *Esrrb* and *Nr0b1*. (**C**) Gene set enrichment analysis using enrichR [26] of the 416 genes with ≥8-fold higher expression in TCM- vs. BM-grown EBs identifies a high enrichment for genes regulated by pluripotency factors OCT4, SOX2 and NANOG in the TF-target interaction database TRRUST [25]. (**D**) Wiggle plots of gene expression data across the indicated pluripotency gene loci of D4 EBs grown in either BM or TCM. *Sox2*, *Esrrb* and *Nr0b1* expression remains far higher in EBs cultured in TCM. ChIP-seq binding sites for the same three factors in ESCs were integrated from previous studies [29,30] and are indicated by the dark blue horizontal bars. (**E**) RT-qPCR expression data of EMT marker genes in EBs at D4 of differentiation. E-Cadherin (*Cdh1*), an epithelial marker, remains more highly expressed in TCM- compared to BM-grown EBs, whereas mesenchymal markers are reduced. Data of six independent biological replicates are normalized to BM conditions and are displayed as mean ± SEM. Statistical analysis was by unpaired two-tailed Student’s *t*-test. *** *p* < 0.001 and **** *p* < 0.0001. (**F**) Expression of *Lif* as the most pertinent pluripotency-promoting factor in two independent TSC lines across a 6-day differentiation time course. Data of two independent biological replicates is normalized to TSC (stem) conditions and are displayed as mean ± SEM. (**G**) LIF protein abundance determined by ELISA and displayed as relative fold-change normalized to total protein levels. Values are displayed as mean ± SEM. Statistical analysis was by one-way ANOVA with Tukey’s multiple comparisons test. * *p* < 0.05.

Having established that TCM impedes cardiomyocyte differentiation significantly in a TSC genotype-dependent manner, we probed the early differentiation delay observed in TCM-exposed ESCs/EBs in more detail. Alkaline phosphatase (AP) staining, a commonly used marker of pluripotency [23,24], of ESCs grown for five days in BM or TCM revealed very few remaining AP-positive colonies in BM conditions, as expected after this extended culture period in the absence of leukemia inhibitor factor (LIF). However, ESCs grown in TCM exhibited a marked retention of AP-positivity in almost one quarter of all colonies (Figure 6A, Supplementary Materials Figure S6A). In line with this observation, the D4 RNA-seq data also showed dramatically higher expression levels of well-known pluripotency genes such as *Oct4* (*Pou5f1*), *Nanog*, *Tet1*, *Esrrb*, *Sox2*, *Nr0b1* and *Zfp42* in TCM-grown EBs compared to those cultured in BM, and these genes remained considerably more highly expressed even at D8 (Figure 6B, Supplementary Materials Figure S6B). Entering the 416 genes that were ≥8-fold more highly expressed in TCM vs. BM into the enrichR functional gene assessment tool revealed a highly significant enrichment of transcriptional regulatory interactions governed by OCT4, SOX2 and NANOG (Figure 6C) [25,26]. From our RNA-seq data it was striking that *Sox2*, *Esrrb* and *Nr0b1* remained particularly highly expressed in TCM-grown EBs, with over 8-fold higher expression levels (Figure 6B). Interestingly, these transcription factors are well known to interact with each other and self-reinforce naïve pluripotency [27,28]. We therefore integrated our RNA-seq data with ChIP-seq data for these three transcription factors [29,30] and found that over half of the genes with ≥8-fold higher expression (222/416) were directly regulated by these three transcription factors, including *Sox2*, *Esrrb* and *Nr0b1* themselves (Figure 6D). Thus, ESCs grown in TCM maintained their pluripotent state for far longer than when cultured in BM, with a particular reinforcement of the *Sox2*, *Esrrb* and *Nr0b1* transcription factor network that in turn also maintains *Oct4*, *Nanog* and *Tet1* expression.

These data were further supported by the observation that TCM-exposed EBs maintained a more pronounced epithelial character, with higher E-Cadherin (*Cdh1*) expression levels and far lower expression of epithelial-mesenchymal transition (EMT) drivers such as *Snai1*, *Snai2*, *Twist1*, *Twist2*, *Zeb1* and *Wnt5* (Figure 6E, Supplementary Materials Figure S7A–C). This lack of induction of EMT progression is in line with the retained features of pluripotency as a result of TCM exposure [31,32]. 

To gain insights into how trophoblast cells may support the maintenance of pluripotency, we investigated gene expression levels of *Lif* and found that TSCs produced substantial amounts of this ESC-required growth factor, with peak production after one day of differentiation and slightly decreasing levels during subsequent differentiation stages (Figure 6F). This time frame of peak *Lif* production directly overlapped with our trophoblast-conditioned medium generation protocol (Figure 1B). *Lif* expression levels at D1 of differentiation were comparable to the production of well-known trophoblast hormones such as placental lactogen 1 (*Prl3d1*) after 5–6 days of differentiation (Supplementary Materials Figure S8A). Moreover, LIF protein abundance was significantly higher in *Pparg* KO TCM and tended to be lower in *Smg9* KO TCM, correlating with the observed changes in cardiac differentiation rates (Figure 5 and Figure 6G). Hence, the prolonged retention of pluripotency features in TCM-exposed ESCs is most likely the result of LIF production by early trophoblast cells.

## 3. Discussion

In this study, we use an optimized differentiation system of murine ESCs into cardiomyocyte-containing EBs to test whether early trophoblast cells produce compounds that may affect these differentiation dynamics. The motivation of this work stems from the hypothesis that trophoblast cells comprising the ExE compartment of the egg cylinder stage mouse embryo are in close physical proximity to the epiblast that would permit for such interactions [2,5,33]. We find that medium conditioned by in vitro cultured TSCs slows down the differentiation process of ESCs into cardiomyocytes, and specifically interferes with early mes-endoderm and cardiac mesoderm specification. Thereby, trophoblast cells affect the rate of cardiac differentiation, a mechanism of regulation that in vivo adds to signals emanating from the VE [34]. Our findings demonstrate that trophoblast cells have a profound influence on the differentiation dynamics of embryonic cells, insofar as they preserve the pluripotent state and delay developmental progression specifically towards the mesoderm lineage.

These insights come on the well-established background that interactions between the in vivo compartments represented by ESCs and TSCs (i.e., the embryonic (ICM, EPI) and trophoblast (ExE) portions of the early conceptus) are critical for embryonic development on multiple levels [2]. ICM and EPI cells are the source of FGF, which is essential to maintain a proliferative, stem-like state in adjacent cells of the ExE in a manner that depends on proximity of ExE cells to the EPI [6]. Insights from various mouse mutant models of FGF/MAPK/ERK pathway components indeed paved the way towards the realization that maintenance of the self-renewal capacity of TSCs in vitro strictly depended on the addition of FGF to the culture medium [6,7,35,36,37,38,39,40]. Conversely, the ExE helps pattern the early mouse embryo, for example through production of BMP4 at the posterior pole of the embryo where these signals are critical to establish primordial germ cells [3]. Trophoblast cells of the ExE also produce enzymes that are required to activate the proform of NODAL into its biologically functional form. Thus, although NODAL is produced by EPI cells, the enzymes required for its activation, FURIN and PCSK6 (PACE4), originate solely from the ExE [41,42,43]. Moreover, trophoblast-produced BMP4 induces *Wnt3* expression in the juxtaposed EPI and VE; WNT, in turn, further activates NODAL [33,42]. The result of these communication systems between the first principal cell lineages of the early mouse conceptus is the establishment of morphogen gradients that help shape the embryo [33,44].

**Figure 7:**
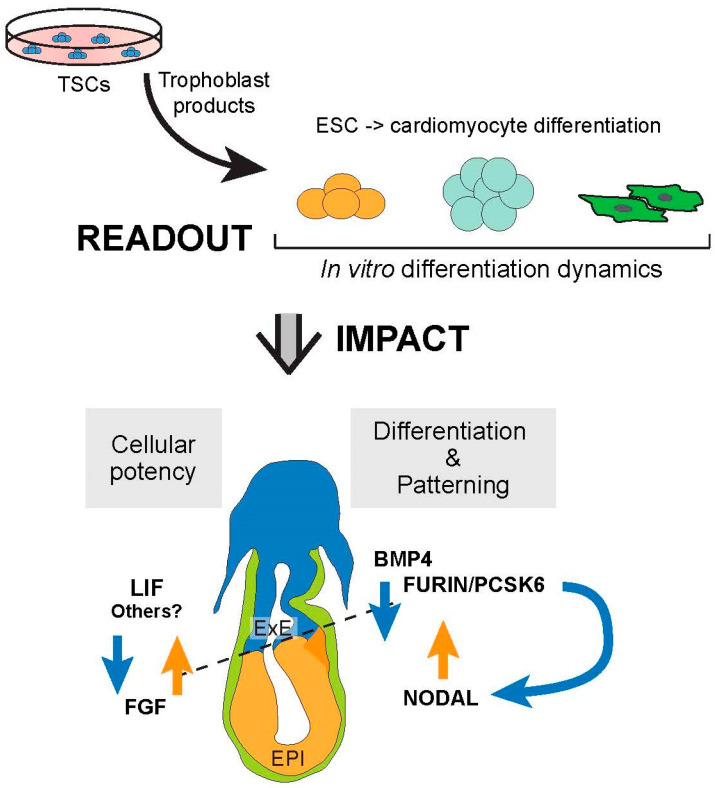
Model of how the current methodology allows to deconstruct the crosstalk between the embryonic and trophoblast compartments using stem cells that reflect these lineages. Thereby, the in vitro system helps inform the complexity of paracrine signaling interactions between the first cell lineages of the early conceptus. EPI = epiblast, ExE = extra-embryonic ectoderm.

Our data reveal that beyond actively governing embryonic lineage differentiation, trophoblast cells also help sustain a pluripotent state in the embryonic compartment. Presumably, this impedance in differentiation rates is necessary to ensure an adequate expansion of progenitor cell pools required for later organogenesis. Normal embryonic development is highly dependent on tightly regulated numbers of precursor cells, and deviations from available progenitor cell pools for subsequent differentiation often has detrimental consequences [45,46,47]. Here, we find that promotion of a stem-like state is not restricted to a unidirectional signal from the embryo to the trophoblast, but that vice versa trophoblast also helps sustain a pluripotent state in embryonic cells (Figure 7). These data are of fundamental importance for the emerging high-profile field of synthetic embryos, as they demonstrate that embryonic development cannot proceed normally without a fully functional trophoblast compartment [48,49].

It is intriguing that the pluripotency markers that are most strongly maintained by trophoblast-produced factors (i.e *Sox2*, *Esrrb* and *Nr0b1*) are exactly those transcription factors that are critical for the self-renewal capacity of both, ESCs and TSCs [29,30]. In TSCs, this network is directly downstream of FGF signaling, with *Esrrb* and *Sox2* being the most acutely down-regulated genes upon MEK inhibition [30]. In ESCs, however, MAPK kinase activation causes differentiation, and the inhibition of this signaling pathway is one of the key components of the so-called “2i” ESC culture conditions that promote ground state pluripotency [50,51,52]. Our data reveal that TSCs promote this shared transcription factor circuit via compartmental interactions in the embryonic lineage. We further show that the maintenance of pluripotency is likely mediated through the production of ample amounts of LIF by trophoblast cells in the stem cell state and, in particular, at early stages of in vitro differentiation. These cellular states are directly comparable to the ExE of the post-implantation embryo [29]. Indeed, ESC differentiation in the presence of exogenous LIF has been reported to inhibit mesoderm formation and the commitment towards cardiomyocytes, thus precisely phenocopying the data we obtained with TCM [53]. Other components secreted or shed by trophoblast cells may also be involved, such as lipid particles or extracellular vesicles. A recent study suggested miR-1249-3p as an important extracellular vesicle-contained miRNA in mediating placentally-directed influences on heart development [54]. Yet, in our hands, we were unable to detect this miRNA in placental samples and did not observe any correlation between the abundance of this miRNA and the phenotype in mouse models with a proven trophoblast-driven impact on heart development (Supplementary Materials Figure S8B) [21]. Moreover, once a functional placenta is established, it is more difficult for trophoblast-produced factors to cross the endothelial cell barrier including its underlying basement membrane to enter the fetal circulation. At this stage of development, the anatomical fine-structure of the placenta and physical distance from the embryo prevents a direct crosstalk through cell–cell interactions or diffusion.

These considerations lend credence to the view that there may be at least two phases in development when trophoblast can affect embryonic development, and this developmental timing dictates the possible modes of interactions. The first phase is during peri-and early post-implantation stages when cell-cell communication is possible across a very short diffusional distance. A second, later phase is after mid-gestation upon formation of a mature placenta when tissue interactions must entail indirect mechanisms and the involvement of additional cell layers, notably the fetal endothelial cells of the placental labyrinth. This concept is underpinned by the various mouse models that collectively have reinforced the existence of a “placenta–heart axis”: of the mutants with a proven placental causality of heart defects, several cause embryonic lethality in the second half of gestation after a structural placenta has formed, and are associated with architectural defects in the placental labyrinth layer. Examples for this scenario are the *Atp11a*, *Ly6e* and *Gpr126* mutations [21,55,56]. Other mutants, however, die much earlier in development around mid-gestation when the placenta is only just forming. This is the case for *Pparg*, *Mapk14* (p38a), *Senp2* and *Rbm15* [9,10,12,57]. Intriguingly, in our in vitro deconstruction of compartmental interactions, *Pparg* KO TCM affected cardiomyocyte differentiation rates, but *Atp11a* KO TCM did not. This is fully consistent with the developmental time line reflected by the EB differentiation system into cardiomyocytes that mirrors the peri-gastrulation phases in development between ~E6-8 when the cardiac crescent emerges and heart development is initiated [47]. Furthermore, our data from the *Smg9* mutant mouse model also suggest that trophoblast-mediated effects on cardiogenesis originate early and are rooted in defects in second heart field formation (Supplementary Materials Figure S5A). Accordingly, we observe substantial differences in our in vitro cardiomyocyte differentiation system with *Smg9* KO TCM compared to WT TCM. 

How precisely these various KOs alter the TCM composition will need to await future investigations, but our data suggest that differences in LIF production by trophoblast may be one critical factor. Indeed, *Pparg*^−/−^ placentas exhibited slightly elevated *Lif* expression levels [58], and we found significantly more LIF protein in *Pparg* KO TCM compared to WT TCM. Higher LIF levels may well explain the exacerbated slowdown in ESC-to-cardiomyocyte differentiation that we observe with *Pparg* KO TCM. Conversely, *Smg9* KO TCM tends to contain less LIF, in line with the accelerated differentiation rates observed with *Smg9* KO TCM. Most importantly, however, our experiments with KO TCMs revealed that the optimized EB differentiation system can sensitively detect early trophoblast-mediated defects, and as such will lend itself to screening protocols for gene mutations that have a trophoblast-rooted impact on early heart formation.

Collectively, our data reveal a reciprocal crosstalk between the trophoblast and embryonic lineages that mutually supports developmental plasticity and slows down differentiation in both compartments. This interaction may be particularly important for the early specification of heart progenitors as the first organ to form after fertilization, thereby providing an explanation for the apparent coupling of placental and cardiac development. Cardiogenesis is extremely sensitive to temporal progression. Developmental acceleration or delay, and commensurate cell number alterations, result in defects in first and/or second heart field formation and consequently lead to cardiac pathologies. Moreover, the pluripotency factors whose expression levels are sustained by trophoblast are directly implicated in regulating the expression of pivotal heart genes, such as *Tbx5*. Our in vitro system demonstrates the importance of a normal trophoblast compartment for the controlled progression of embryonic cell differentiation towards the cardiac lineage, and establishes a powerful tool to identify the nature of the various types of interactions between the first two committed cell lineages in intricate detail.

## 4. Materials and Methods 

### 4.1. ESC and TSC Lines Used and Standard Culture Conditions

The mouse embryonic stem cells (ESCs) used in this study were the R1 cell line [59], which are of XY genotype, stably transfected with a cardiac-specific α-myosin heavy chain (αMHC, *Myh6*) promoter-driven enhanced green fluorescent protein (GFP) construct. These cells were generously provided by the laboratory of Dr. James Cross, University of Calgary. These cells harbored a *Hand1*-null mutation [60,61] and were used because a *Hand1*-null background significantly elevates cardiomyocyte differentiation rates, with an apparent default mesoderm differentiation pathway towards a cardiomyocyte fate [62]. The enhanced rate of cardiomyocyte formation was particularly useful in our approaches where cardiomyocyte differentiation was suppressed or delayed, which increased the power of our experiments as it prevented that results were based on differences in very few GFP-positive cells only. For the most pertinent data (delay in differentiation, cardiac mesoderm induction and retention of pluripotency genes), experiments were also performed with wild-type R1 ESCs, yielding comparable outcomes (shown in the Supplementary Information). The ESCs were routinely cultured in Dulbecco’s modified Eagle’s medium (DMEM; 319-015-CL, Wisent Inc., Saint-Jean-Baptiste, QC, Canada) containing 15% fetal bovine serum (12483-020, ThermoFisher Scientific, Waltham, MA, USA), 1× non-essential amino acids (11140-050, ThermoFisher Scientific, Waltham, MA, USA); 1 mM sodium pyruvate (600-110-EL, Saint-Jean-Baptiste, QC, Canada), 1× antimycotic/antibiotic (450-115-EL, Saint-Jean-Baptiste, QC, Canada) and LIF (500 U/mL, Cambridge Stem Cell Institute, Cambridge, UK).

For the vast majority of experiments, wild-type mouse trophoblast stem cells (TSCs) were the TS-Rs26 line (XY sex), a kind gift of the Rossant lab, Toronto, Canada. TSCs were routinely maintained as described previously [7,63]. TSCs mutant for *Atp11a*, *Smg9* and *Pparg* were derived by CRISPR-Cas9 mediated exon deletion on the TS-Rs26 background and were described previously [21]. *Ssr2* KO TSC clones were generated by deleting exons 1 and 2 of the main protein-coding isoform *Ssr2*_201 and were confirmed by PCR genotyping. CRISPR gRNA sequences were: UP1-TAAAACGAAATAAGCTGCAT, UP2-GAAGAAATTGGAGGACACAC, DOWN1-TGTTTCTTGTCATATATCTG, DOWN2-CATTGCCTGGAAAACATCAA. At least two independent KO clones per gene were used for conditioned medium generation. For analysis of *Lif* expression levels, the TS-EGFP TSC line (XX sex) [7] was used in addition to TS-Rs26; this line was also a kind gift of the Rossant laboratory.

### 4.2. Conditioned Medium Generation

For the generation of trophoblast-conditioned medium, TSCs were plated at a defined cell density of 10,000 cells/cm^2^ in 10 cm dishes in 15 mL base medium (BM) containing 15% fetal bovine serum (098150, Lot 450115189, Wisent Inc., Saint-Jean-Baptiste, QC, Canada) in DMEM (319-015-CL, Wisent Inc., Saint-Jean-Baptiste, QC, Canada), 1 mM sodium pyruvate (600-110-EL, Wisent Inc., Saint-Jean-Baptiste, QC, Canada), 1× antimycotic/antibiotic (450-115-EL, Wisent Inc., Saint-Jean-Baptiste, QC, Canada) and 50 µM 2-mercaptoethanol (21985-023, ThermoFisher Scientific, Waltham, MA, USA). Three days later, the supernatants were harvested, filtered and stored in 40 mL aliquots at −20 °C as trophoblast-conditioned media (TCM). TCM was carefully checked for having the same pH as BM. TCM media was used diluted 50:50 with BM. 

### 4.3. Embryoid Body Differentiation

To initiate ESC differentiation (D0), aggregation of ESCs was achieved via microwell forced-aggregation in Aggrewell™ 400 plates (34415, Stem Cell Technologies, Vancouver, BC, Canada), [18,64]. The cell seeding density was 600 cells per individual microwell, with 1200 microwells per well in 24 well plates. Thus, 720,000 cells in 1.8 mL BM or TCM were added to each 24-well well. The ESCs were then incubated in the wells for 48 h with partial media change after 24 h. At D2, the resulting embryoid body (EB) aggregates from each well were transferred to suspension culture in sterile 100 × 15 mm bacteriological grade polystyrene Petri dishes (Fisherbrand FB0875731, ThermoFisher Scientific, Waltham, MA, USA) and maintained in 15 mL medium (BM or TCM) on a rotary orbital shaker (Orbitron rotary II, model 260250, Marshall Scientific, Hampton, NH, USA) at 25rpm and tilt angle of 4.5 degree.

At D4, EBs were plated in 1.2 ml medium on 24-well plates pretreated with 0.2% gelatin at a density of 15 EBs/well, and the medium was changed every day. For observation and histological analysis, some EBs were kept on Petri dishes (3D culture, on shaker) with media change on D5. Green fluorescence of cells and EBs was visualized under an Olympus CKX53 inverted tissue culture microscope coupled to a CoolLED pE-300 (Quorum Technologies Inc, Puslinch, ON, Canada) ight source.

### 4.4. Alkaline Phosphatase Staining

The alkaline phosphatase (AP) staining as a hallmark of pluripotency was performed as described previously [65]. Briefly, ESCs were plated at a density of 1000/cm^2^ on 0.2% gelatin-coated tissue culture plates and cultured in BM or TCM. Five days after plating, cells were fixed and the alkaline phosphatase staining was performed using 5-bromo-4-chloro-3-indolyl phosphate-nitro blue tetrazolium kit (S3771, Promega, Madison, WI, USA) according to the manufacturers protocol. Colonies were imaged and scored by morphology and level of AP staining. Colonies of tightly packed and flattened AP-positive cells were counted as undifferentiated and colonies of mixtures of unstained and stained cells or entirely unstained cells with flattened morphology were considered mixed or differentiated, respectively. Three independent experimental replicates were performed.

### 4.5. RNA Extraction and RT-qPCRs

Total RNA was extracted using either TRI reagent (T9424, Sigma, St. Louis, MO, USA) or the RNeasy Mini Kit (74106, Qiagen, Hilden, Germany) according to the manufacturer’s protocols. 1–2 µg of total RNA was used for cDNA synthesis with RevertAid H-Minus reverse transcriptase (EP0451, ThermoFisher Scientific, Waltham, MA, USA) and a mix of oligo-d(T)_18_ (FERSO132, ThermoFisher Scientific, Waltham, MA, USA) and random primers (FERSO142, ThermoFisher Scientific, Waltham, MA, USA). Quantitative (q)PCR was performed using the QuantiFast (25057, Qiagen, Hilden, Germany), QuantiNova (208057, Qiagen, Hilden, Germany) or SsoAdvanced Universal SYBR Green Supermix (1725274, BioRad, Hercules, CA, USA) and, where possible, intron-spanning primer pairs on a QuantStudio 3 (ThermoFisher Scientific, Waltham, MA, USA), CFX96 or CFX384 (BioRad, Hercules, CA, USA) real-time PCR thermocycler. All primers were tested for efficiency and correct product amplification prior to use. Normalized expression levels are displayed as mean relative to the BM control or WT TCM control, as indicated; error bars indicate standard error of the means (S.E.M.) of at least three independent biological replicates. All primer sequences are provided in Supplementary Materials Table S1.

### 4.6. RNA-Sequencing

The RNA for transcriptomic analysis was isolated using the RNeasy Mini Kit (74106, Qiagen, Hilden, Germany). Library construction with NEB Ultra II Directional RNA Library Prep kitv (#E7765, New England Biolabs, Ipswich, MA, USA) and sequencing on an Illumina NovaSeq 6000 (50 bp paired-end) was completed by the University of Calgary’s Centre for Health Genomics and Informatics facility. The reads were aligned with STAR to the mouse genome (GrCM38/mm10) and count tables were constructed with htseq-count (23104886, 25260700) resulting in 36M-52M gene counts per sample. Differential gene expression and sample visualization were completed in R version 3.4.3 and 4.1.0 (URL https://www.R-project.org/, (accessed between 1 January 2022 and 30 June 2023)). Briefly, differentially expressed genes were detected in both DESeq2 and EdgeR, had a false discovery rate < 0.1, and a fold change > |1.5|. Mapped sequencing data were also visualized with the SeqMonk software v1.48 (URL https://www.bioinformatics.babraham.ac.uk/projects/seqmonk/ (accessed between 1 January 2022 and 30 June 2023)) to integrate ChIP-seq data and to produce wiggle plots.

### 4.7. Determination of LIF Protein Levels in TCM

The LIF protein levels were determined by ELISA (ab238261, Abcam, Cambridge, UK) according to the manufacturer’s protocol and performed in technical duplicate for every WT, *Smg9* KO and *Pparg* KO TSC clone (n = 3 each). Values were normalized to total protein amounts determined by BCA protein assay (23225, ThermoFisher Scientific, Waltham, MA, USA).

### 4.8. Statistical Analyses

For statistical analyses of RT-qPCR and ELISA data, Student’s *t*-test or ANOVA was performed as indicated to calculate statistical significance (*p* < 0.05) using GraphPad Prism software version 9.5. All details of these statistical analyses are reported in the figure legends.

## Data Availability

RNA-seq data have been deposited in the GEO database with accession number: GSE236001.

## Supplementary Materials

The supporting information can be downloaded at: https://www.mdpi.com/article/10.3390/ijms241512423/s1.
