# Supplementary material for: The Trophoblast Compartment Helps Maintain Embryonic Pluripotency and Delays Differentiation towards Cardiomyocytes
Source: Int J Mol Sci. 2023 Aug 4;24(15):12423. doi: 10.3390/ijms241512423 (PMC10418709; doi:10.3390/ijms241512423)
Supplement: Supplementary file 1 [file ijms-24-12423-s001.zip › Supplementary Information_R1.pdf]

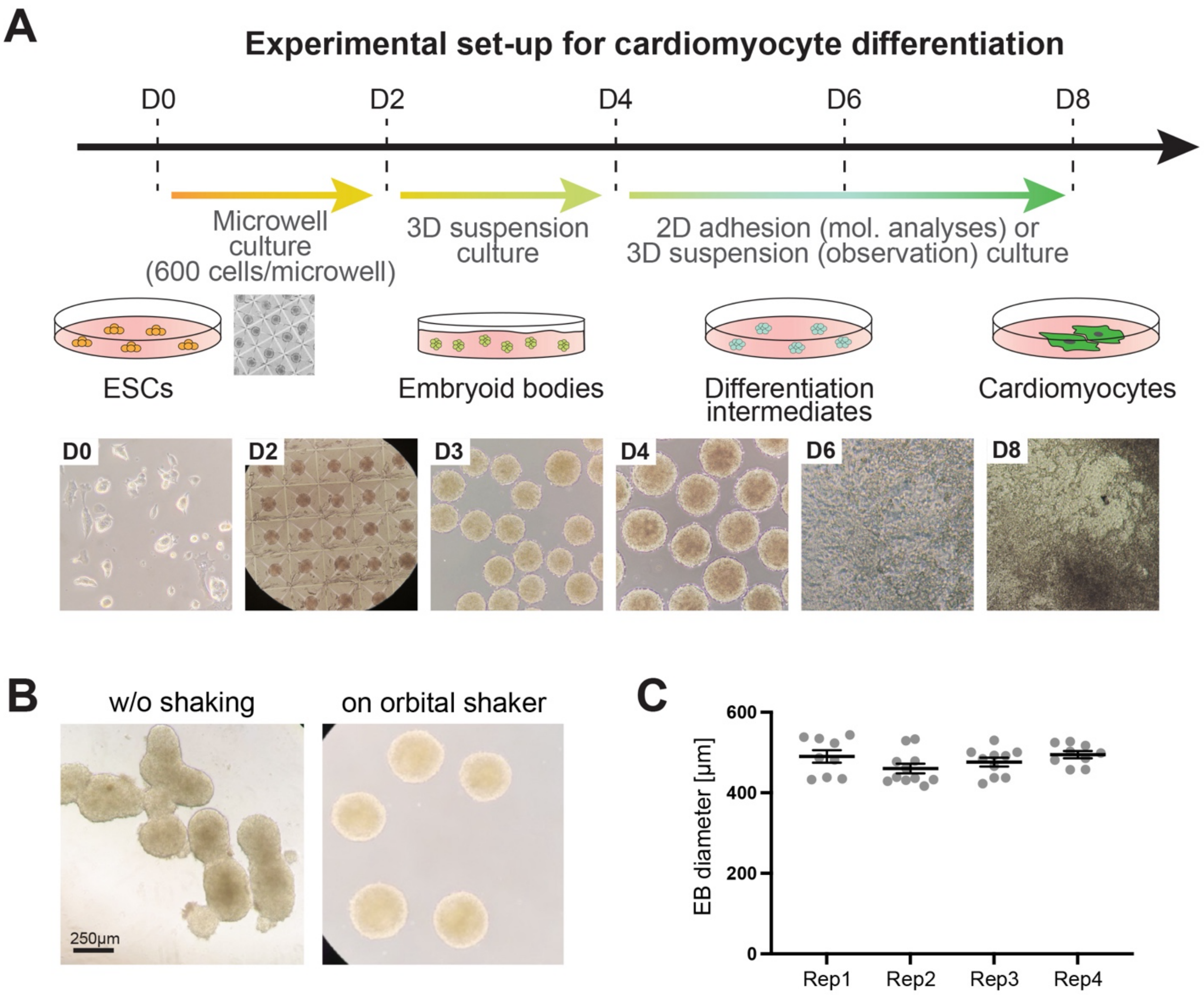


**Suppl. Figure S1.** Optimized embryoid body (EB) differentiation protocol to study the effects of trophoblast-conditioned medium on cardiomyocyte differentiation from ESCs.

(A) Schematic depiction of the formation of EBs in microwells after plating of defined cell numbers, followed by suspension and then adhesion culture (for molecular analyses) to promote differentiation of ESCs into beating cardiomyocytes. For visualization purposes of the progression of cardiomyocyte differentiation, EBs were left in suspension culture for the remainder of the time course.

(B) Photographs of EBs cultured for 4 days in suspension culture, with and without the use of an orbital shaker. EBs are exquisitely uniform in size following our protocol using the shaker.

(C) Assessment of EB diameters after 7 days of differentiation in suspension culture. EB sizes are very uniform within and between replicate experiments. Values are displayed as mean ± SEM.

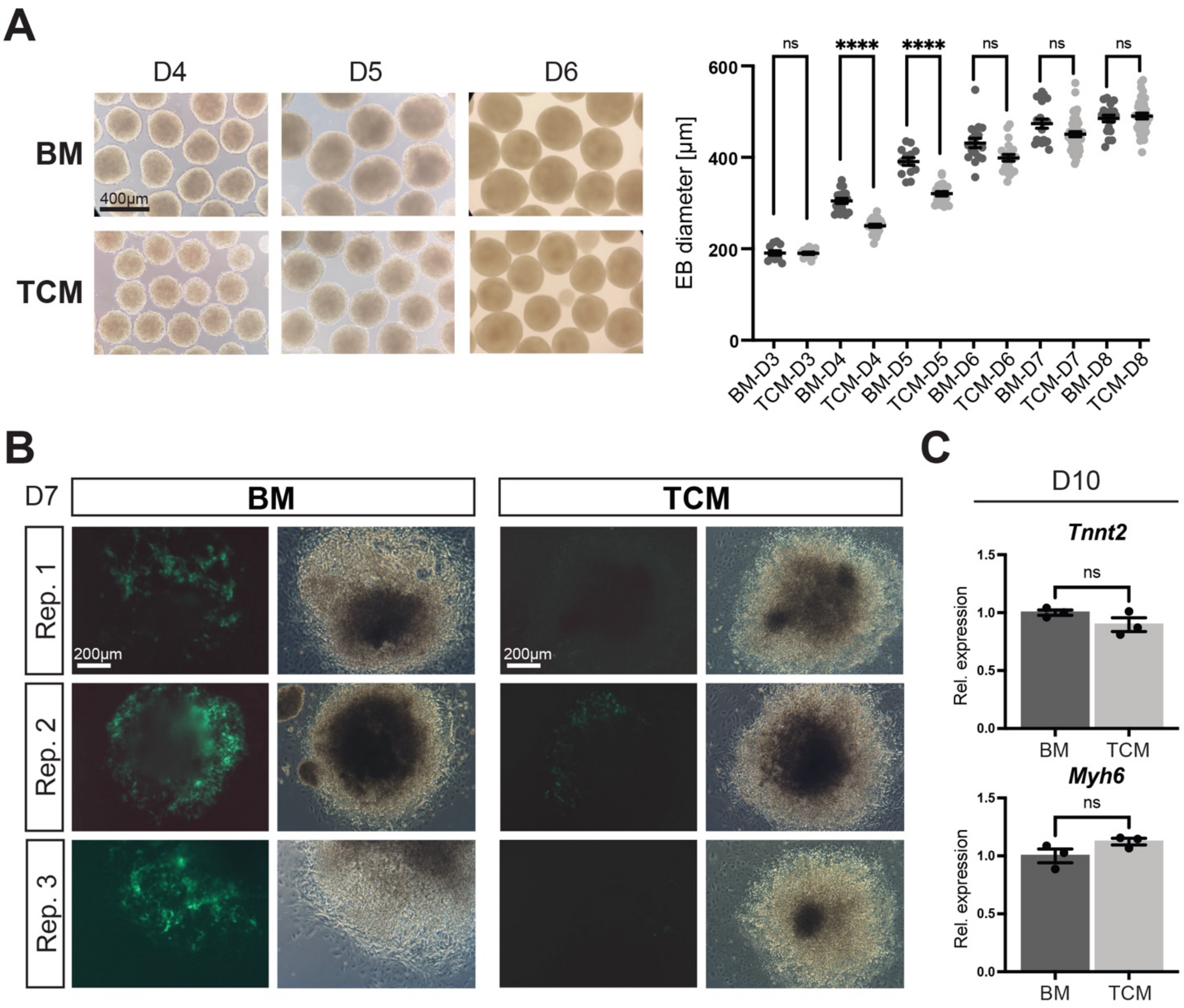


**D**

Assessment of beating EB numbers

| Condition | D6 | D8 | D9 |
|---|---|---|---|
| **BM** | 71% (22/31) | 100% (60/60) | Whole well moving |
| **TCM** | 0% (0/45) | 12% (7/60) | Some spots/areas beating |
| **BM -> TCM** | 81% (30/37) | 100% (50/50) | Whole well moving |
| **TCM -> BM** | 2.5% (1/40) | 7% (4/60) | Some spots/areas beating |

**Suppl. Figure S2.** TCM impacts the first phase of ESC differentiation towards cardiomyocytes.

(A) Photographs of EBs grown in suspension culture and assessment of EB sizes (diameters) across an 8-day differentiation time course. BM- and TCM-grown early EBs at day 3 (D3) are of the same size (dark grey and light grey points, respectively), but on D4 and D5 BM-cultured EBs start to expand more quickly. By D6, TCM-grown EBs catch up with BM-cultured EBs. Data are from 2-4 independent biological replicate experiments per day and condition, with 5-17 EBs measured for

each experiment. Values are displayed as mean ± SEM. Statistical analysis was by one-way ANOVA with Tukey's multiple comparisons test. **** $p<0.0001$.

(B) Cardiomyocyte reporter *Myh6*-driven green fluorescence of attached EBs in adhesion culture conditions is visible by day 7 (D7) in BM-cultured cells, whereas the vast majority of cells is still GFP-negative when cells are grown in TCM.

(C) RT-qPCR of EBs at D10 of differentiation for the mature cardiomyocyte markers *Tnnt2* and *Myh6*. Data are of three independent biological replicates normalized to BM conditions and displayed as mean ± SEM. Statistical analysis was by unpaired two-tailed Student's t-test. ns = non-significant.

(D) Evaluation of the fraction of beating EBs grown in adhesion culture conditions at D6, D8 and D9 of the ESC-to-cardiomyocyte differentiation process depending on early or late exposure to TCM, as detailed in Fig. 2A. The frequency of beating EBs confirms that TCM has the most profound impact during the first 4 days of differentiation.

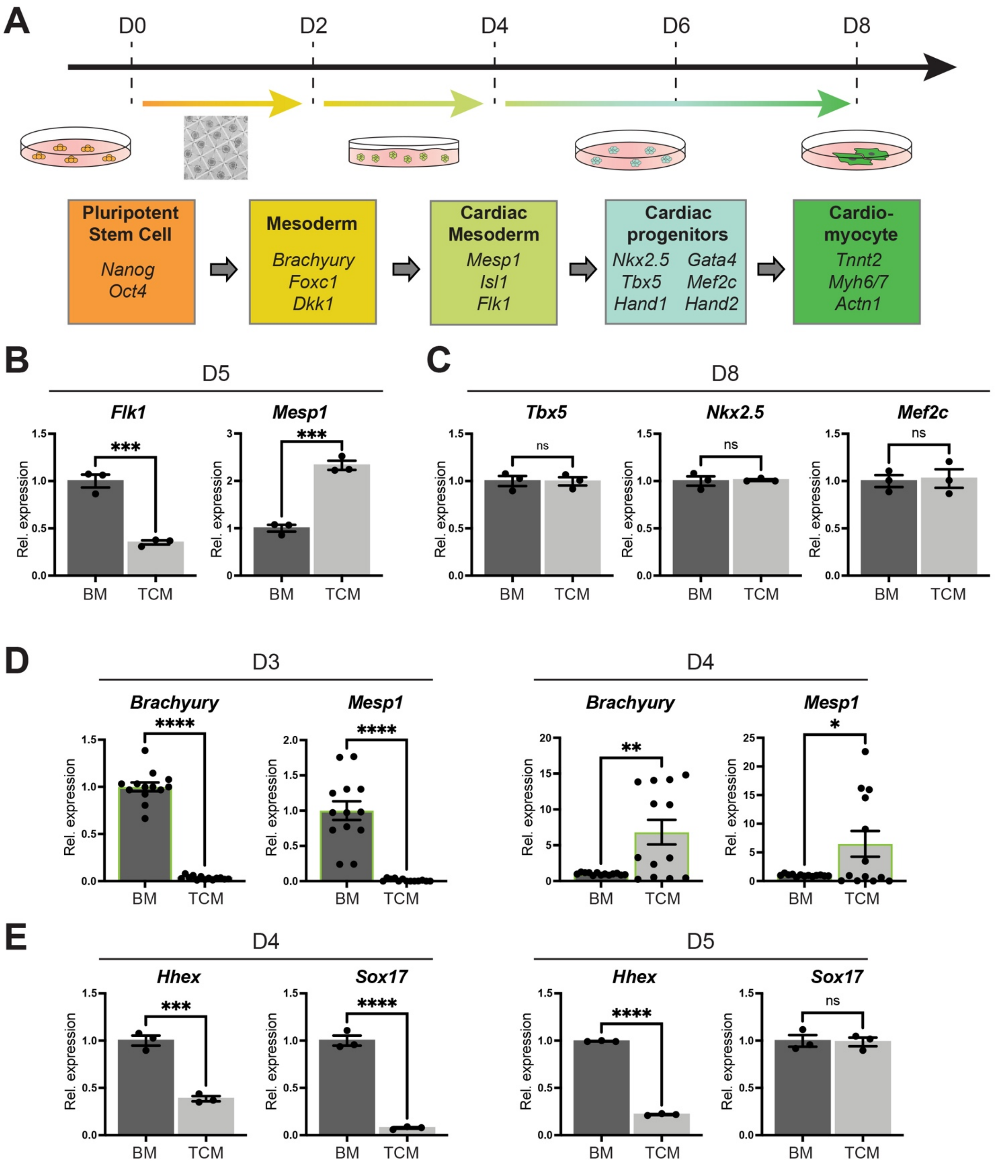


**Suppl. Figure S3.** TCM delays mesoderm and specifically cardiac mesoderm differentiation.

(A) Schematic depiction of the progression of differentiation from ESCs to cardiomyocytes including representative marker genes.

(B) RT-qPCR analysis of additional marker genes at D5 of differentiation. By this time, *Mesp1* expression in BM-cultured EBs has declined while that in TCM-grown EBs is increasing. *Flk1* up-regulation is still lagging behind in TCM-grown EBs.

(C) RT-qPCR analysis of cardiac progenitor marker genes at D8 of differentiation. After the initial differentiation bottleneck, TCM-grown EBs "catch up" in the progression of differentiation with no remaining differences in cardiac progenitor markers *Nkx2.5* and *Mef2c* at D8.

(D) RT-qPCR analysis of the critical differentiation bottleneck-defining markers in the wild-type R1 ESC line, producing comparable results except that the activation of *Brachyury* and *Mesp1* peaks around 1 day earlier than in the *Hand1*-null R1 ESC line used throughout. Data are of 13 independent biological replicates normalized to BM conditions and displayed as mean ± SEM. Statistical analysis was by unpaired two-tailed Student's t-test. * $p<0.05$, ** $p<0.01$, **** $p<0.0001$.

(E) RT-qPCR analysis of mes-endoderm genes *Hhex* and *Sox17* that are requisite for *Mesp1* activation and that are also delayed in their activation in TCM-exposed EBs. Data in (B), (C) and (E) are of three independent biological replicates normalized to BM conditions and displayed as mean ± SEM. Statistical analysis was by unpaired two-tailed Student's t-test. *** $p<0.001$, **** $p<0.0001$.

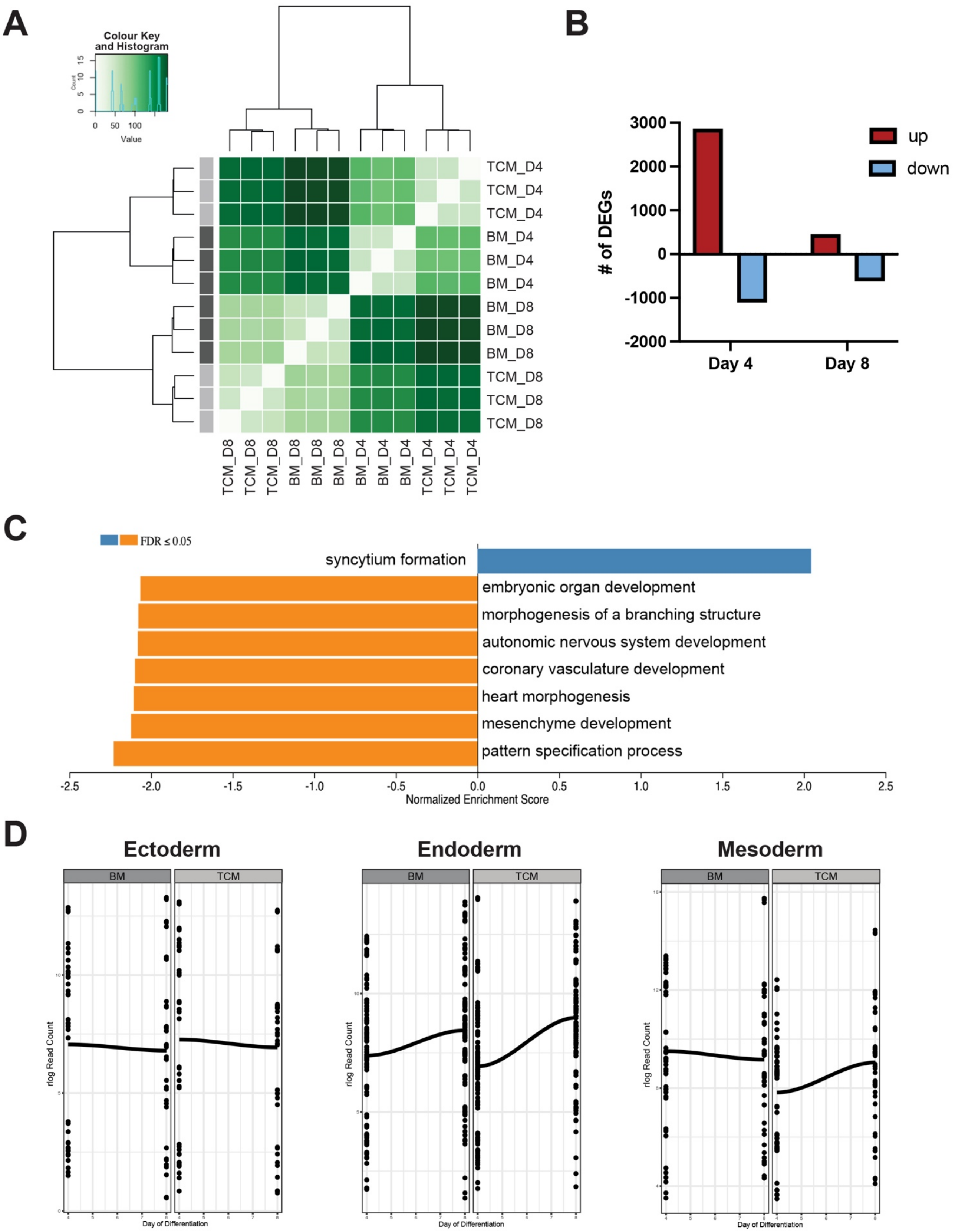
A
Colour Key and Histogram
Count
Value
TCM_D4
BM_D4
BM_D8
TCM_D8
B
# of DEGs
up
down
Day 4
Day 8
C
FDR ≤ 0.05
syncytium formation
embryonic organ development
morphogenesis of a branching structure
autonomic nervous system development
coronary vasculature development
heart morphogenesis
mesenchyme development
pattern specification process
Normalized Enrichment Score
D
Ectoderm
Endoderm
Mesoderm
BM
TCM
rlog Read Count
Day of Differentiation

**Suppl. Figure S4.** Transcriptomic comparison of BM- and TCM-grown EBs.

(A) Sample-to-sample heatmap of dissimilarity plot based on Euclidean distances, visualizes the extremely close similarity between biological replicates, providing proof for the robustness of the EB differentiation protocol (Suppl. Fig. 1) (white=identical, dark green= most dissimilar).

(B) Number of up- and down-regulated genes in D4 and D8 EBs grown in BM vs TCM (>|1.5| FC and FDR <0.05, DESeq2 and EdgeR).

(C) Gene enrichment analysis (WebGestalt, non-redundant biological process) identifies gene ontology terms related to heart morphogenesis as significantly depleted in genes identified as down-regulated in TCM-grown EBs.

(D) Germ layer marker gene expression between D4 and D8 of EB differentiation plotted as the regularized logarithm (rlog) of read counts of RNA-seq data. Ecto- and endoderm differentiation is not or only marginally affected by TCM, whereas mesoderm differentiation is delayed in TCM-grown vs. BM-grown EBs, as shown by the trendlines.

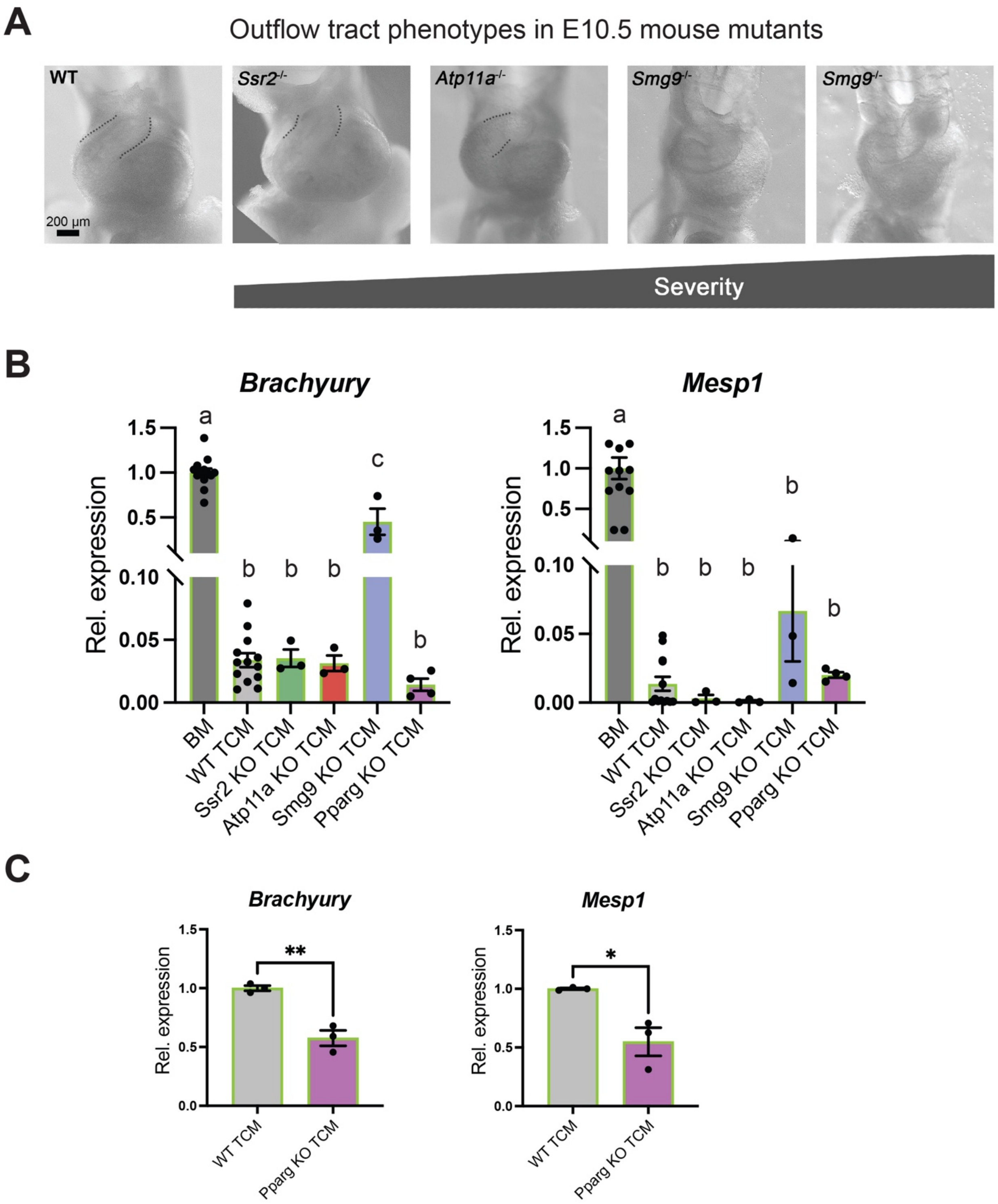


**Suppl. Figure S5.** Outflow tract defects in E10.5 *Ssr2*$^{-/-}$, *Atp11a*$^{-/-}$ and *Smg9*$^{-/-}$ embryos and differentiation dynamics in WT ESCs.

(A) Heart morphology of E10.5 mouse embryos of the indicated genotypes shows deficiencies in outflow tract length and rotation (dotted lines) in all of the mutant embryos, but the severity of this

phenotype is by far the most pronounced in *Smg9* mutants. These phenotypes are indicative of defects in second heart field formation which originate 2-3 days earlier.

(B) RT-qPCR analysis of wild-type R1 ESC-derived EBs at D3 grown in BM or TCM from wild-type (WT) or various mutant TSCs. Identical to the data obtained with the *Hand1*-nulll R1 ESCs, TCM generated from mutant TSCs for genes that have an early impact on cardiogenesis *in vivo* (*Smg9*, *Pparg*) alter EB differentiation rates compared to WT TCM, in particular *Smg9* KO TCM. By contrast, conditioned medium from TSCs mutant for genes that either do not have a trophoblast effect on heart development *in vivo* (*Ssr2*) or that exhibit a later gestational impact of the placenta on heart formation (*Atp11a*) have no divergent effect from WT TCM. Data are representative of three biological replicates per KO TCM, each performed with their internal BM and WT TCM controls. Values are normalized to BM and are displayed as mean ± SEM. Statistical analysis was by one-way ANOVA with Tukey's multiple comparisons test. The letters above bars denote the outcome of statistical comparisons, with statistically significant differences ($p < 0.05$) indicated by discrepant letters, whereas identical letters indicate $p>0.05$.

(C) Comparative analysis of the effects of *Pparg* KO TCM vs WT TCM on wild-type R1 ESC-derived EBs differentiation dynamics assessed by RT-qPCR for informative marker genes at D3. *Pparg* KO TCM exacerbates the differentiation-inhibiting effect seen with WT TCM. Data are of 3 independent biological replicates normalized to WT TCM conditions and are displayed as mean ± SEM. Statistical analysis was by unpaired two-tailed Student's t-test. * $p<0.05$, ** $p<0.01$.

A

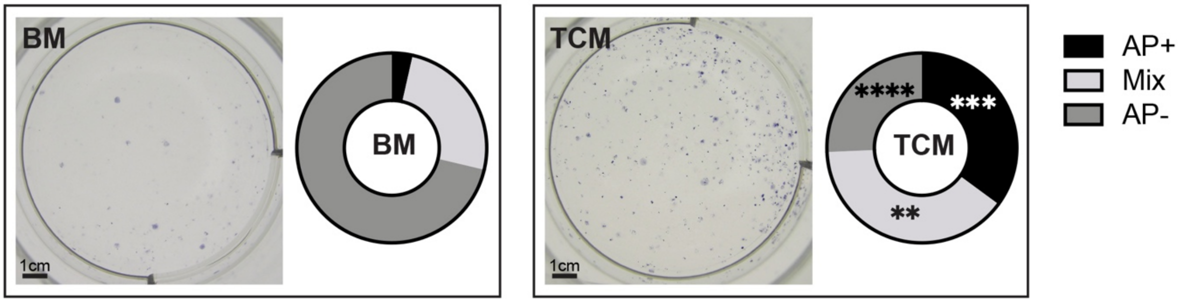


B

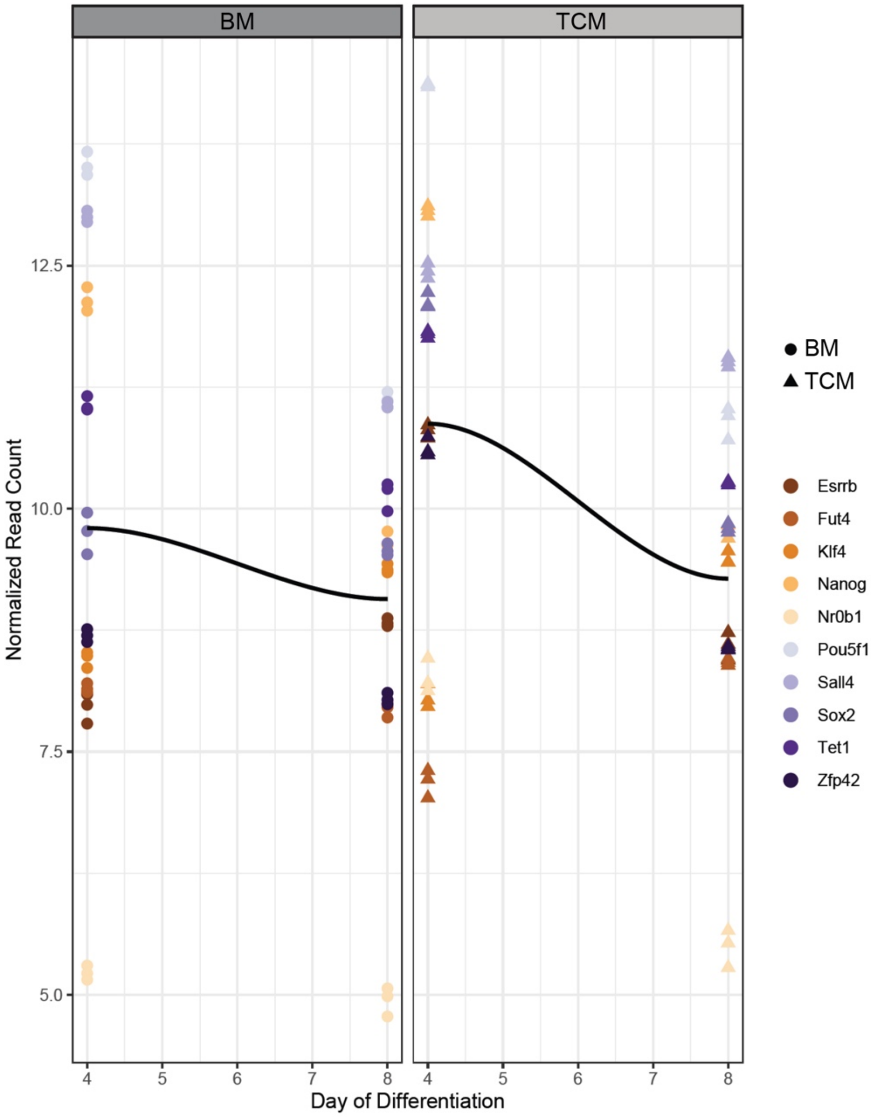


**Suppl. Figure S6.** TCM prolongs pluripotency features in ESCs and EBs.

(A) Alkaline phosphatase (AP) staining as a pluripotency readout on wild-type R1 ESCs after five days in BM or TCM. The number of AP-positive colonies is dramatically higher in TCM conditions. Data

are from three independent biological replicates. Statistical analysis was by multiple unpaired Student's t-tests with Holm-Šídák multiple comparisons test. ** $p<0.01$, *** $p<0.001$, **** $p<0.0001$.

(B) Dynamics of pluripotency gene expression between D4 and D8 of EB differentiation plotted as the regularized logarithm (rlog) of read counts of RNA-seq data (circles and triangles). Pluripotency gene expression is higher at D4 and stays higher at D8 in TCM-grown vs. BM-grown EBs, as shown by the trendline (black).

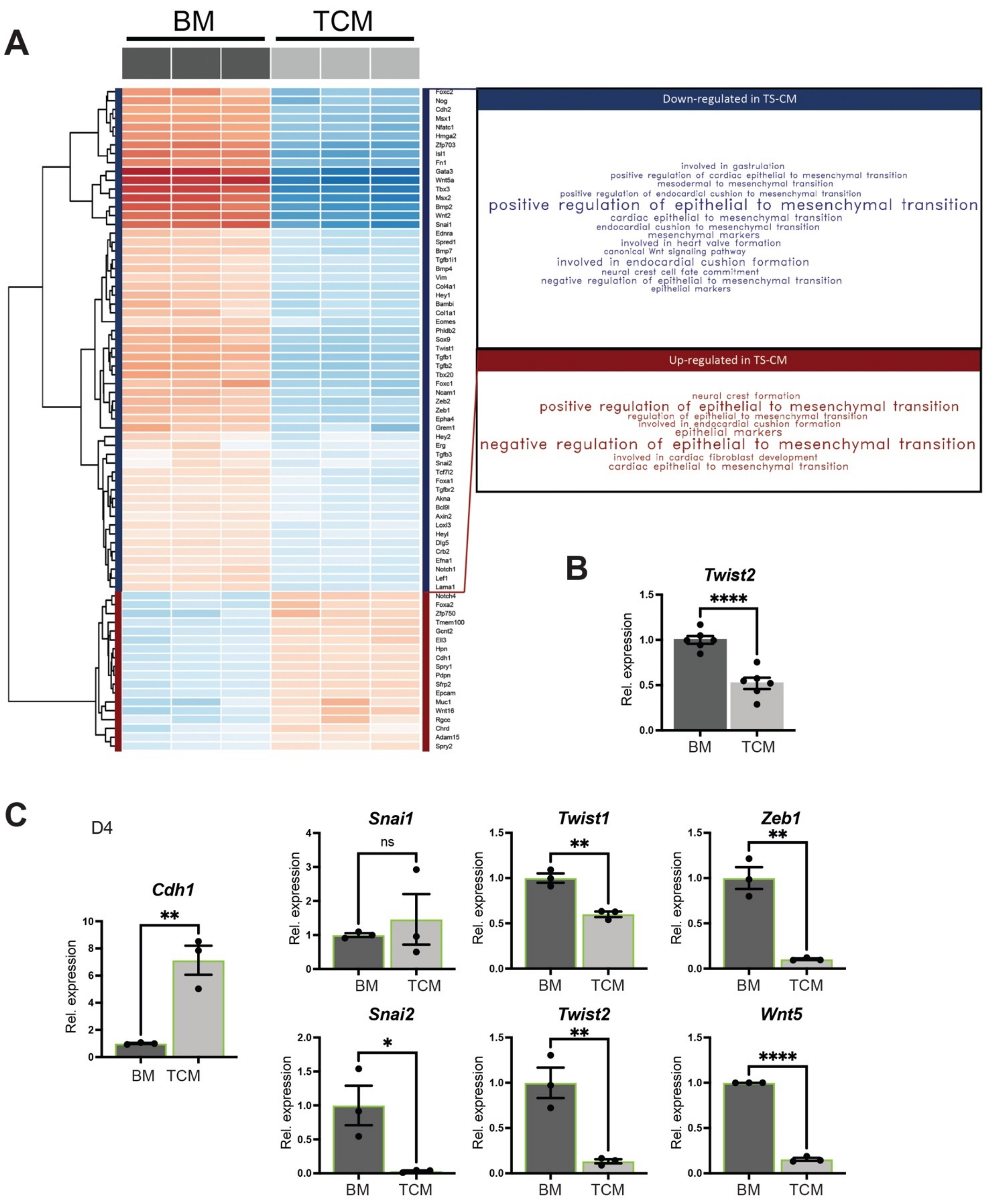


**Suppl. Figure S7.** TCM suppresses epithelial-mesenchymal transition (EMT).

(A) Dendrogram of gene variance and heatmap of EMT marker genes (GO:0001837), using rlog-centred RNA-seq reads (logFC >|0.3|), demonstrate two main clusters of up or down-regulated

genes. Word clouds were generated for each cluster using gene functions obtained from MGI (annotated terms and context), with size proportional to the frequency of that function within the gene cluster. These word clouds show that down-regulated genes in TCM-grown EBs are associated with driving EMT progression, whereas up-regulated genes suppress EMT transition.

(B) RT-qPCR analysis of *Twist2*, am additional marker of EMT progression in *Hand1*-null EBs at D4. Samples are from Fig. 6E.

(C) RT-qPCR expression data of EMT marker genes in D4 EBs derived from wild-type R1 ESCs. E-Cadherin (*Cdh1*), an epithelial marker, remains more highly expressed in TCM- compared to BM-grown EBs, whereas mesenchymal markers are reduced. Data of three independent biological replicates are normalized to BM conditions and are displayed as mean ± SEM. Statistical analysis was by unpaired two-tailed Student's t-test. * $p<0.05$, ** $p<0.01$, **** $p<0.0001$.

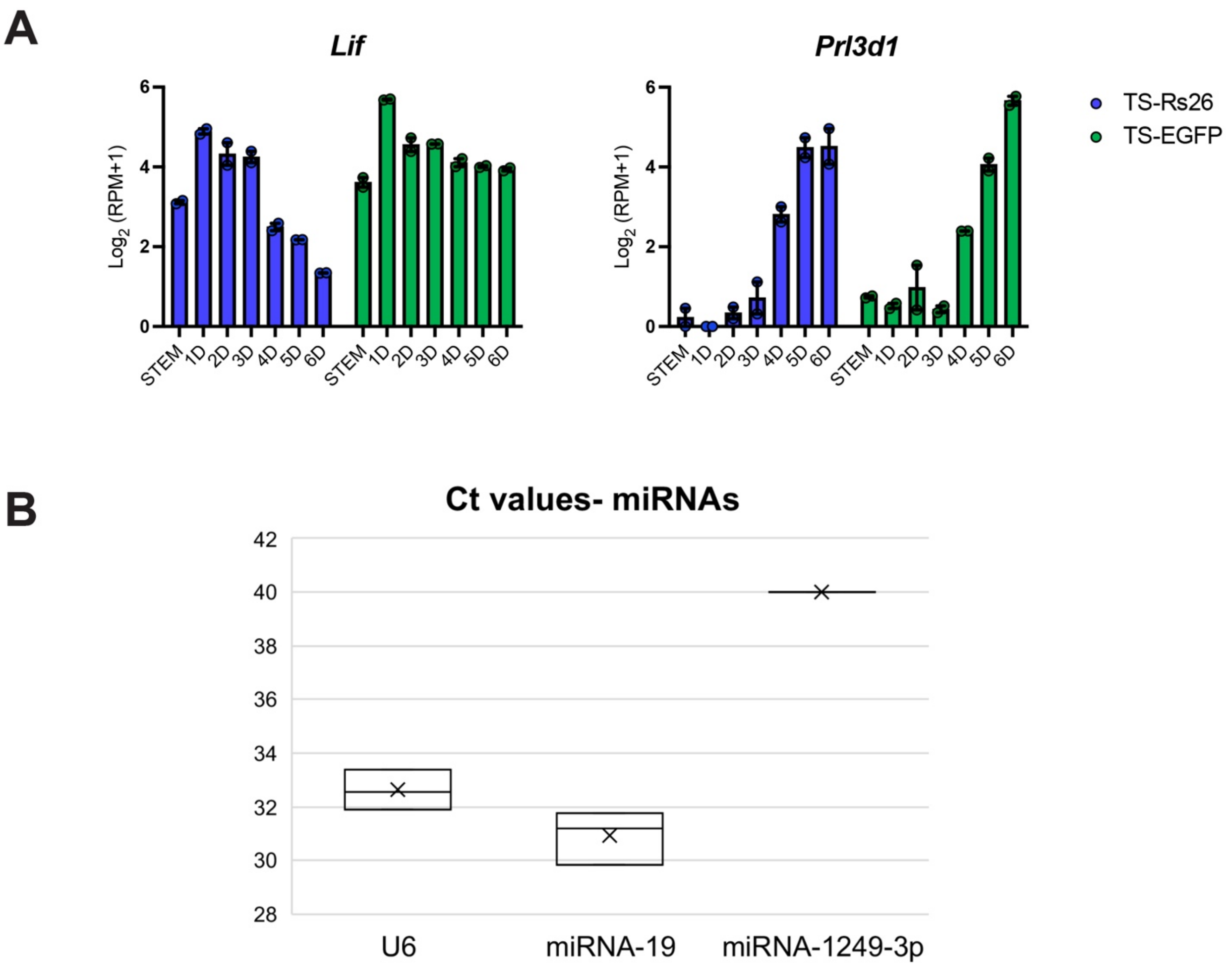


**Suppl. Figure S8.** Expression of relevant growth factors, hormones and miRNAs in TSCs and placentas.

(A) Comparative expression analysis of *Lif* and placental lactogen 1 (*Prl3d1*), a key placental hormone, in two different TSC cell lines across a differentiation time course. Graphs show two independent biological replicates from RNA-seq data normalized to library size ($Log_2$ (RPM+1)).

(B) Real-time PCR Ct values of U6 and miRNA-19 control miRNAs, and miRNA-1249-3p, from E14.5 wild-type placentas. miRNA-1249-3p has been recently implicated in mediating placental influences on heart development. cDNA was obtained from total RNA (mirVana kit AM1560, Invitrogen, Waltham, MA, USA) using the QuantiMir Kit (RA420A-1, SBI Systems Biosciences, Palo Alto, CA, USA). Data are from three biological replicates, performed in triplicate each. miRNA-1249-3p was not detectable and Ct values were set to total cycle numbers, 40. The horizontal lines of the box plot demarcate individual values, the “x” the mean,

**Supplementary Table S1. List of primers used**

| | |
|---|---|
| Brachyury-F | TGCCTGTGAGTCATAACGCC |
| Brachyury-R | ACCATTGCTCACAGACCAGA |
| Cdh1-F | GCTCTCATCATCGCCACAGA |
| Cdh1-R | TTCGAGGTTCTGGGATGGGA |
| Flk1-F | AAGGAGTCTGTGCCTGAGAACTG |
| Flk1-R | GAGAAAATCGCCAGGCAAAC |
| Gata4-F | AATCTAAGACGCCAGCAGGT |
| Gapdh-F | ACCCAGAAGACTGTGGATGG |
| Gapdh-R | CACATTGGGGGTAGGAACAC |
| Gata4-R | CTGCTGTGCCCATAGTGAGA |
| Hhex-F | ACCTGGTTTCAGAATCGCCG |
| Hhex-R | GTGTCCAAACTGTCCAACGC |
| Lif-F | GGAAGCAGAGGGGCCCAACAAC |
| Lif-R | ATACTGGAGCCGTGGTCTCGCA |
| Mef2c-F | AGATACCCACAACACACCACGCGCC |
| Mef2c-R | ATCCTTCAGAGAGTCGCATGCGCTT |
| Mesp1-F | TGTACGCAGAAACAGCATCC |
| Mesp1-R | TTGTCCCCTCCACTCTTCAG |
| Myh6-F | GATGGCACAGAAGATGCTGA |
| Myh6-R | CTGCCCCTTGGTGACATACT |
| Nkx2.5-F | TTACCCGGGAGCCTACGGTG |
| Nkx2.5-R | GCTTTCCGTCGCCG CCGTGCGC |
| Snai1-F | GAAAGGCCTTCTCTAGGCCC |
| Snai1-R | GTTGGAGCGGTCAGCAAAAG |
| Snai2-F | AGCTCCACTCCACTCTCCTT |
| Snai2-R | GAAGTGTCAGAGGAAGGCGG |
| Sox17-F | CGAGCCAAAGCGGAGTCTC |
| Sox17-R | TGCCAAG GTCAACGCCTTC |
| Tbx5-F | CCTCCGGCTTTCCTGCTAAG |
| Tbx5-R | ATCTGTATCGGCCATGGTGC |
| Tnnt2-F | TGGTCCTGATGAAGAAGCCA |
| Tnnt2-R | GCACCAAGTTGGGCATGAAG |
| Twist1-F | CTCGGACAAGCTGAGCAAGATT |
| Twist1-R | ATTTTCTCCTTCTCTGGAAACA |
| Twist2-F | CGTGCCATCCACCTCCG |
| Twist2-R | AGCCACAAGGTTGTCCAGG |
| Wnt5a-F | AGGGTTCCTATGAGAGCGCA |
| Wnt5a-R | GGAGCCAGACACTCCATGAC |
| Zeb1-F | AGTGCACTGAATGCGGGAAG |
| Zeb1-R | TTCGGGCATTCGTATGGCTT |
